# IL1RAP is an immunotherapeutic target for normal karyotype triple-mutated acute myeloid leukemia

**DOI:** 10.1186/s40364-025-00769-z

**Published:** 2025-04-14

**Authors:** Arnaud Métois, Marie-Eve Bordeleau, Louis Theret, Azadeh Hajmirza, Ossama Moujaber, Jean-François Spinella, Jalila Chagraoui, Nadine Mayotte, Isabel Boivin, Éric Audemard, Léo Aubert, Véronique Lisi, Banafsheh Khakipoor, Azer Farah, Éric Bonneil, Alma Robert, Julie Lippens, Anna Moraitis, François Béliveau, Albert Feghaly, Geneviève Boucher, Richard Marcotte, Patrick Gendron, Pierre Thibault, Sébastien Lemieux, Guillaume Richard-Carpentier, Vincent-Philippe Lavallée, Josée Hébert, Philippe P. Roux, Guy Sauvageau

**Affiliations:** 1 The Leucegene Project at Institute for Research in Immunology and Cancer, Université de Montréal, Montréal, Québec H3 T 1 J4 Canada; 2 Centre Hospitalier Universitaire Sainte-Justine Research Center, Montréal, Québec H3 T 1 C5 Canada; 3 Medical Devices Research Center, National Research Council Canada, Montréal, Québec H4P 2R2 Canada; 4 Quebec Leukemia Cell Bank, Maisonneuve-Rosemont Hospital, Montréal, Québec H1 T 2M4 Canada; 5 Human Health Therapeutic Research Center National Research Council Canada, Montréal, Québec H4P 2R2 Canada; 6 Department of Chemistry, Faculty of Arts and Science, Université de Montréal, Montréal, Québec H3 T 1 J4 Canada; 7 Department of Biochemistry and Molecular Medicine, Faculty of Medicine, Université de Montréal, Montréal, Québec H3 T 1 J4 Canada; 8 Princess Margaret Cancer Centre, University Health Network, Toronto, ON M5G 2 C1 Canada; 9 Department of Medicine, Division of Medical Oncology and Hematology, Temerty Faculty of Medicine, University of Toronto, Toronto, ON M5S 1 A8 Canada; 10 Department of Pediatrics, Faculty of Medicine, Université de Montréal, Montréal, Québec H3 T 1 J4 Canada; 11 Hematology and Oncology Division, Centre Hospitalier Universitaire Sainte-Justine, Montréal, Québec H3 T 1 C5 Canada; 12 Department of Medicine, Faculty of Medicine, Université de Montréal, Montréal, Québec H3 T 1 J4 Canada; 13 Division of Hematology-Oncology, Maisonneuve-Rosemont Hospital, Montréal, Québec H1 T 2M4 Canada; 14 Department of Pathology and Cell Biology, Faculty of Medicine, Université de Montréal, Montréal, Québec H3 T 1 J4 Canada

**Keywords:** IL1RAP, Acute myeloid leukemia, Surfaceome, Immunotherapy, Cancer, Single cell

## Abstract

**Background:**

Surface antigens of potential clinical significance remain under-characterized in AML. The European Leukemia Network classifies normal karyotype AML (NK-AML) mutated for NPM1 (NPM1c) as a distinct entity associated with favorable outcomes if not associated with FLT3-ITD mutation. A subset of NPM1c NK-AML shows additional mutations in 2 genes: FLT3 (FLT3-ITD) and DNMT3 A. These leukemias, also referred to as NK triple mutated AML (NKt-AML), are particularly difficult to eradicate with current treatment options. Therefore, novel therapies are necessary that use proteins specifically expressed at the surface.

**Methods:**

In order to identify surface antigens for immunotherapy in NKt-AML, an extensive multi-omic analysis was conducted on primary AML samples. Surface proteome enrichment was performed on 100 primary AML samples, twelve of which were NKt-AML. Transcriptome analysis was carried out on the 691 primary AML samples, and single-cell RNA sequencing was conducted on 23 primary AML samples.

**Results:**

Herein, using multi-omics data from the Leucegene collection, we identify IL1RAP as a promising antigen for this AML subgroup. We demonstrate that IL1RAP is expressed at the surface of primitive AML cells reminiscent of leukemic stem cells in NKt-AML primary human AML specimens, while showing relatively low expression levels in normal bone marrow HSCs. Furthermore, results indicate that elevated IL1RAP expression associates with poor overall and relapse-free survival in the Leucegene cohort of AML patients and predicts nonresponse to hematopoietic stem cell transplantation. Finally, we show that IL1RAP protein is internalized following exposure to specific antibodies, suggesting that IL1RAP represents an interesting target for antibody–drug conjugate development in NKt-AML.

**Conclusions:**

IL1RAP exhibits preferential expression within NKt-AML, correlating with diminished overall survival rates and diminished responsiveness to hematopoietic stem cell transplantation. Moreover, internalization of IL1RAP presents a promising avenue for immunotherapeutic intervention.

**Supplementary Information:**

The online version contains supplementary material available at 10.1186/s40364-025-00769-z.

## Introduction

Acute myeloid leukemia (AML) is characterized by a multitude of mutations and chromosomal aberrations such as translocations, inversions and deletions. These characteristics are routinely integrated to classify this disease in several cytogenetic/genetic subgroups [1]. Most specimens in each of these subgroups present 2 additional levels of heterogeneity. First, genetic subclones are the norm rather than the exception for each patient (oligoclonal disease). Second, subclones typically vary in their differentiation level. Leukemic stem cells (LSCs), the most primitive leukemia cell type contributing to AML onset and relapse, lie at the apex of this hierarchy, and mature blasts at the bottom, along with intermediate cellular constituents between these 2 extremes. Single-cell RNA-sequencing experiments have been most informative at revealing this cellular hierarchy [2–5]. Interestingly, accumulating evidence indicates that both genetic alterations and cell type dictate treatment response for each AML subset [6, 7].

Normal karyotype triple-mutated AML (or NKt-AML) is an AML subtype associated with poor clinical outcome, with a 5-year overall survival rate below 20% and represents 6% to 10% of AML cases [8, 9]. It displays a high LSC frequency and a GRP56 hi/CD34 lo immunophenotype [10, 11]. NKt-AML is characterized by a normal karyotype and by the co-occurrence of mutations in three specific genes: NPM1, DNMT3 A and FLT3. NPM1 is a nuclear chaperone, known to shuttle proteins and nucleic acids between nucleus and cytoplasm. It is involved in ribosomal biogenesis by promoting the nuclear export of 5S rRNA and its incorporation into 60S ribosomal subunits [9], hence impacting cell growth. Additionally, NPM1 interacts with p53 to regulate its stability and transcriptional activity [9, 12, 13]. NPM1 is one of the most frequently mutated gene in NK AML (50% of NK AML cases), with the insertion of 4 nucleotides in the 3’end of the mature transcript observed in more than 95% of NPM1-mutated cases and leading to a neomorphic protein with aberrant cytoplasmic localization (NPM1c) [9, 14, 15]. In NK and NKt-AML, this mutation is associated with elevated expression of numerous genes such as HOXA/B, MEIS1 and PBX3, which have been reported to mediate maintenance of the leukemic state for this specific AML subset [9]. DNMT3 A is a DNA methyltransferase involved in hematopoietic stem cell program repression during cellular differentiation via the downregulation of HSC-specific genes [16]. DNMT3 A loss of function mutation R882H, occurring in 30% of NK AML, is the most frequent DNMT3 A mutation in AML (50% of DNMT3 A-mutated cases) and is associated with poor prognosis in hematological cancers, possibly through its impact on leukemia stem cell proliferation and apoptosis [17–20]. Finally, FLT3 is a receptor tyrosine kinase expressed by immature bone marrow (BM) hematopoietic cell populations and that regulates cell survival, proliferation and differentiation, when stimulated by FLT3 ligand [21]. FLT3 mutations occur in two specific forms: point mutations affecting the FLT3 kinase domain and internal tandem duplications (ITD) in the juxta-membrane domain. FLT3-ITD is the most relevant FLT3 aberration in AML as it confers poor prognosis when associated with NPM1c mutation [8, 22, 23] and is mutated in 45.7% of NK AML cases [24].

The current standard of care for NKt-AML treatment includes induction chemotherapy with the 7 + 3 regimen with the addition of the FLT3 inhibitor Midostaurin based on the prolonged overall survival reported for FLT3-mutated AML treated with Midostaurin compared to control patient group [25]. Complete remission rates did not significantly differ between the 2 groups, however [25], suggesting that additional therapeutic strategies are needed for FLT3-mutated and NKt-AML patients. In addition to chemotherapy, many immunotherapeutic approaches are currently under development in AML. Indeed, several antigens are currently being targeted in clinical trials by antibodies inducing antibody-dependent cell cytotoxicity (ADCC), antibody–drug conjugates (ADCs), and more recently by chimeric antigen receptor T cell (CAR-T cells) and BiTE antibodies [26–29]. Gemtuzumab ozogamicin (Mylotarg TM), the only therapeutic antibody approved by the U.S. Food and Drug Administration produces significant survival improvement for favorable cytogenetic risk AML (inv(16) and t(8;21), however, limited benefit is observed for adverse cytogenetic risk patients [30]. One major issue associated with the targeting of cell surface antigens in a therapeutic context in AML is the shared expression of antigens between LSCs and normal hematopoietic stem cells (HSPCs). Accordingly, it becomes critical to identify antigens specifically overexpressed by most primitive AML cell populations, including LSCs, compared to their normal counterpart. Technologies such as single-cell RNA sequencing can be exploited to explore the makeup of these antigens at high resolution in multiple AML and normal bone marrow cell subpopulations including most primitive cells.

As part of the Leucegene program, our group developed a multiomics approach involving transcriptomics, genomics, cytogenetics, proteomics, and chemical screening to characterize AML subgroups and develop new therapies for this disease. This approach was exploited for the characterization of the 691 primary human AML specimens of the Leucegene collection. This endeavor led to the identification of AML subgroup-specific gene expression profiles, new functional biomarkers, novel mutations in AML subgroups and several previously uncovered therapeutic targets [10, 11, 31–46]. More recently, our group developed a comprehensive approach combining surface proteomics, RNA sequencing and single-cell RNA-sequencing to characterize the AML surfaceome through the analysis of primary human AML specimens [47]. This effort enabled the identification of several AML antigens common to all AML subgroups analyzed, as well as potential LSC markers and provided an overview of antigens expressed by specific AML subgroups. Additionally, our group developed SPAT (Surface protein annotation tool: https://spat.leucegene.ca) [48], and LASA (Leucegene AML surfaceome atlas: https://lasa.leucegene.ca/lasa_v2/)[47], allowing for surface proteome, transcriptome and single-cell transcriptome analysis of primary human AML specimens of the Leucegene cohort. Herein, using these datasets and tools as well as newly generated data, we report the in depth surfaceome analysis of NKt-AML and identify IL1RAP (Interleukin 1 Receptor Accessory Protein) as a promising antigen for this AML subgroup that shows elevated expression in primitive AML cell subpopulations and limited expression in normal BM HSCs. Importantly, elevated IL1RAP expression associates with adverse outcome in the Leucegene cohort and identifies AML patients refractory to current therapeutic strategies. IL1RAP protein is an essential component of the Interleukin 1 receptor complex, inducing the signaling cascade of interleukin. This complex plays a major role in immune response to infection, tissue damage and stress by triggering the synthesis of proinflammatory protein [49]. Elevated expression of IL1RAP has been documented in many type of cancer including solid tumors (pancreatic ductal adenocarcinoma, Ewing sarcoma, gliomas, triple negative breast cancer, non-small cell lung cancer, stomach adenocarcinoma, or cervical cancer), and hematological malignancies (CML and AML) [50, 51]. Despite evidence demonstrating high IL1RAP expression in AML, its correlation with specific cytogenetic/genetic subgroups remains unclear. This information is essential for determining future diagnostic and therapeutic strategies. Prior research has indicated that IL1RAP can be targeted therapeutically to eliminate leukemia; however, the blocking activity of certain IL1RAP antibodies is ineffective in the context of FLT3-ITD. [52–54]

## Methods

### Primary human AML specimens and AML subgroup classification

The Leucegene AML surfaceome cohort is composed of 100 specimens, 61% male and 39% female, with a median age at diagnosis of 59 years. The Leucegene AML RNA sequencing cohort is composed of 691 specimens, 58% male and 42% female, with a median age at diagnosis of 57 years. The use of primary human AML specimens for this study was approved by the Research Ethics Boards of Université de Montréal and Maisonneuve-Rosemont Hospital. Leukemia primary specimens used in this work were collected with informed consent. Sample characterizations were performed by the Quebec Leukemia Cell Bank after obtaining an institutional Research Ethics Board–approved protocol. The Quebec Leukemia Cell Bank is a biobank certified by the Canadian Tissue Repository Network [47].

AML specimens were classified based on WHO 2022 classification guidelines [55] and mutational, cytogenetic characteristics. Cytogenetic aberrations and karyotypes were described according to the International System for Human Cytogenomic Nomenclature 2020 [56]. Complex karyotype was defined as leukemia with 3 or more clonal chromosomal abnormalities in the absence of one of the recurrent genetic abnormalities: t(8;21), inv(16) or t(16;16), t(9;11), t(v;11), t(6;9), inv(3) or t(3;3), and AML with BCR-ABL1 [57]. Other AML primary specimens were classified as inv (16), CK, t(8;21), MECOM-r, del(5q), mono 7, del(7q), tri/tetra 8, t(6;9) or KMT2 A-r AML according to their cytogenetic information. NK triple-mutated AML was defined as normal karyotype AML with co-occurrent NPM1, DNMT3 A and FLT3-ITD mutations. RUNX1-mutated and NPM1-mutated AML were defined as normal or intermediate abnormal karyotype AML with respective RUNX1 mutation and NPM1-mutation.

### Datasets

Mass spectrometry data from the Leucegene AML surfaceome cohort have been previously published [47] and deposited to the ProteomeXchange Consortium via the PRIDE [58] partner repository with the dataset identifier PXD043772, PXD044480 and 10.6019/PXD044480. Single-cell RNA sequencing data generated from primary human AML specimens used in this manuscript previously published [47] and deposited in the GEO repository (GSE241989). Leucegene RNA sequencing data from primary human AML specimens (*n* = 691) and normal human hematopoietic cell populations are available in the GEO repository (GSE67040 and GSE98310). Immunohistochemistry data from normal human tissues used for this manuscript were extracted from the Human Protein Atlas [59] Portal (proteinatlas.org). The human platelet membrane proteomics mass spectrometry dataset was previously published [60]. Human cell atlas normal bone marrow single-cell RNA sequencing data from eight healthy donors was obtained from the Human Cell Atlas. [61] Therapeutic antibody information was extracted from the Thera-SAbDab database (opig.stats.ox.ac.uk/webapps/newsabdab/therasabdab) on 23rd of March 2023 [62].

### Surface proteomics and single-cell RNA-sequencing

Surface proteomic and single-cell RNA-sequencing data generation from primary human AML specimens has been described elsewhere [47]. The proteome mapping was performed on review dataset from UniProt v2023_04. Differential analysis was performed without imputation and maximum 4 missing value per condition, using DEP.

### Additional single-cell RNA-sequencing data analysis

Processing and analysis of scRNA-seq were performed in R4.2 and Python 3 using Seurat v4.3 [63] and Scanpy [64]. All samples are processed individually using log-normalization with a 10,000-scale factor, most variable identification, and PCA reduction method [65]. Finally, samples were integrated according to the Seurat pipeline [66]. Integration of multiple samples is made using 2000 most variable features across all samples. Dimension reduction is performed using 50 dimensions for the PCA, and UMAP. UMAP is computed on the “integrated” assay and using the “umap-learn” method with the following parameters: n.epoch = 500 and seed = 123. All single-cell figures are plotted using SCP, using the normalized data. Cell types within each specimen were annotated using an artificial neural network-based classifier developed by our group (available on GitHub https://github.com/lavalleelab/ANN-CAST). ANN-CAST was trained with labeled single-cell RNA-sequencing data obtained from healthy donors available from the Human Cell Atlas. It labels each cell independently according to the most similar normal cell type.

### Flow cytometry

IL1RAP was labeled using anti-IL1RAP coupled with PE (phycoerythrin) from R&D Systems (clone #89,412 cat #FAB676P). In addition, cells were stained with CD45 (clone #HI30 BDbioscience cat#560,777) and GPR56 (clone #CG4 biolegend cat#358,206). Data acquisition was performed on FACSFortessa (BD bioscience). Bone marrow flow cytometry analysis was done using same IL1RAP antibody and CD14 (clone #HCD14 Biolegend cat#325,620) and CD45RA (clone #HI100, cat#563,870). Anti-CD34 is used for bone marrow sorting. Cells are stained for 20 min in the dark and washed two times with 5 mL of PBS.

Flow cytometry results were analyzed using FlowJo V10 software. Dead cells and debris were excluded with FSC (discriminating cell size) and SSC (discriminating cell granularity) signals. The blast population is defined according to CD45 mid expression. IL1RAP positive population was determined using CD45 hi lymphocyte population, known to be IL1RAP negative. The gating strategy is confirmed using FMO (Fluorescence Minus One) to avoid measuring background signals.

### Cell lines

OCI-AML3 cell lines were provided by the University Health Network (Toronto). Cells were expanded in aMEM supplemented with 20% heat-inactivated FBS and incubated at 37 °C, 5% CO2.

### Internalization assay

To assess if IL1RAP is a potential target for antibody drug-conjugated (ADC), we assess IL1RAP internalization. We used the OCI-AML3 cell line to test IL1RAP internalization. Cells were stained at t = 0 with anti-IL1RAP (PE clone #89,412 cat #FAB676P from R&D Systems) and incubated at 37 °C in aMEM supplemented with 20% FBS-Hi. Secondary staining was performed at different time points (t = 0, t = 15 min, and t = 30 min) with an anti-PE (APC clone # PE001 cat# 408,107 from Biolegend) antibody. Mean fluorescence intensity is measured by flow cytometry.

### Indirect ADC inhibition assay

AML- 5 and U266 cells were seeded at the appropriate cell densities in complete media in white, opaque bottom, tissue-culture treated 384 well plates (Costar # 3570) and incubated for 24 h at 37 °C and 5% CO2. Anti-mouse Fc region antibodies (a mix of 1:1 of anti mIgG1 and anti mIgG2a) or anti-human Fc hIgG1, conjugated to PNU (secondary Ab) were used to link the specific anti-IL1RAP antibodies (respectively mouse or human) to the drug. This mixture, at a 1:1 molar ratio of primary to secondary antibody, was incubated for 30 min at room temperature in DPBS prior to serial dilution (when applicable) and antibody application to the cells. In the case of AML- 5 cells, which express FcγR, an Fc blocking reagent (hIgG from Sigma, # I4506) was incubated with the cells for 1–2 h at room temperature prior to addition of the primary + secondary antibody mixes.

Following 5 days of exposure to primary and secondary antibody mixes, cell viability was assessed using a CellTiter-Glo® luminescence ATP detection kit (Promega Corporation). Signal output was measured on a luminescence plate reader (Envision, Perkin Elmer) set at an integration time of 0.1 s. Each concentration point (S) was normalized to signal in the negative control, untreated wells (NC) and expressed as % survival (S/NC X 100). When dose–response curves were performed, potency (IC50) and efficacy (% maximum inhibition) were calculated from a non-linear 4-PL curve fit of the % survival versus log of the concentrations (GraphPad Prism software).

### Statistical analyses and data visualization

All figures were generated with ggplot2 on R v4.4 or Seaborn packages on python v3.10.4. Wilcoxon test is used to calculate the p-value.

## Results

### NKt-AML overexpresses a distinct subset of cell surface proteins

**Fig. 1:**
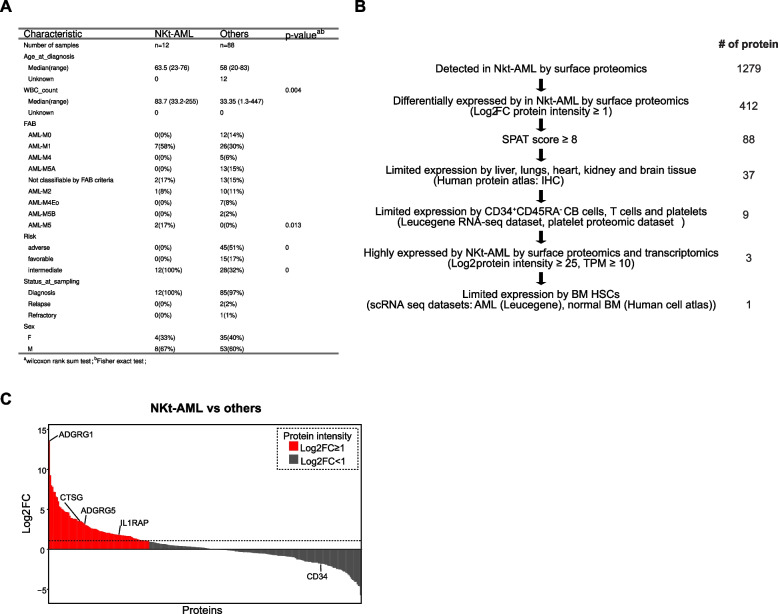
NKt-AML surfaceome enrichment and pipeline analysis. **A** Leucegene AML surfaceome cohort characteristic comparison between NKt-AML and other primary human AML specimen. **B** Schematic representation of NKt-AML antigen selection pipeline. **C** Comparison of protein intensity between NKt-AML (*n* = 12) and other (*n* = 88) specimens of the Leucegene AML surfaceome cohort. FC: fold change in protein intensity. Red bars represent proteins with log_2_FC ≥ 1. Highlighted are candidate antigens (CTSG, ADGRG5, IL1RAP) and proteins overexpressed (ADGRG1) or underexpressed (CD34) by NKt AML

To characterize the surfaceome of NKt-AML and identify antigens for immunotherapeutic targeting, we exploited proteomic data from the Leucegene AML surface proteome dataset (*n* = 100 primary human AML specimens), which includes 12 NKt-AML specimens. Patient characteristics from this cohort are shown in Fig. 1A (see also Table S1) [47]. All specimens in the NKt-AML group and 97% of samples from control group were sampled at initial diagnosis, and mostly exclude relapse and refractory states. White blood cell counts were significantly higher in the NKt-AML group than in the control group, likely reflecting the association of this feature with FLT3-ITD mutation [67]. A total of 1279 proteins were detected in NKt-AML specimens by mass spectrometry (Fig. 1B) [68], and differential surface proteome analysis revealed that among those, 412 are overexpressed by NKt-AML compared to other AML subgroups (Log2 FC in protein intensity ≥ 1, Fig. 1B, and Fig. 1C (red bars)). To highlight cell surface proteins from this subset, we used the SPAT algorithm (Surface Protein Annotation Tool), developed by our group, that exploits publicly available annotations and proteomic datasets to compute a score that estimates the likelihood of plasma membrane localization for a given protein [48]. 88 proteins showed a SPAT score above or equal to 8, the confidence threshold for surface protein identification determined by SPAT, likely corresponding to bona fide cell surface proteins [48](Table S2). As a proof of concept for this approach, GPR56 (ADGRG1), a surface marker reported to define NKt-AML leukemic stem cell, was found to be the surface protein most significantly and differentially expressed by NKt-AML (Fig. 1C). Additionally, CD34, shown to be poorly expressed by NKt-AML, was underexpressed by NKt-AML compared to specimens from other AML subgroups (Fig. 1C) [11]. Together, these results demonstrate that NKt-AML selectively expresses a subset of cell surface proteins compared to other AML subgroups and prompted further characterization of these candidates for antigen identification.

To identify antigens for immunotherapeutic targeting, the 88 candidates were subjected to expression analysis using public datasets to select proteins with limited expression in normal essential organ tissues (lungs, liver, brain, kidney and heart, immunohistochemistry data from The human protein atlas) [59], platelets (platelet proteomics dataset) [60] and hematopoietic cells (expression ≤ 10 TPM in cord blood CD34 + CD45RA- cells and peripheral blood T cells, Leucegene normal human hematopoietic cell RNA-sequencing dataset [43]), while showing relatively high expression levels in NKt-AML (TPM ≥ 10) (Fig. 1B, Fig. S1, Table S3). Expression thresholds in normal essential organ tissues and hematopoietic cells were based on an analysis of the expression profile of antigens currently targeted by therapeutic antibodies in phase II and III clinical trials or approved by the FDA previously reported by our study [47].

### IL1RAP is a novel cell surface antigen for NKt-AML

**Fig. 2:**
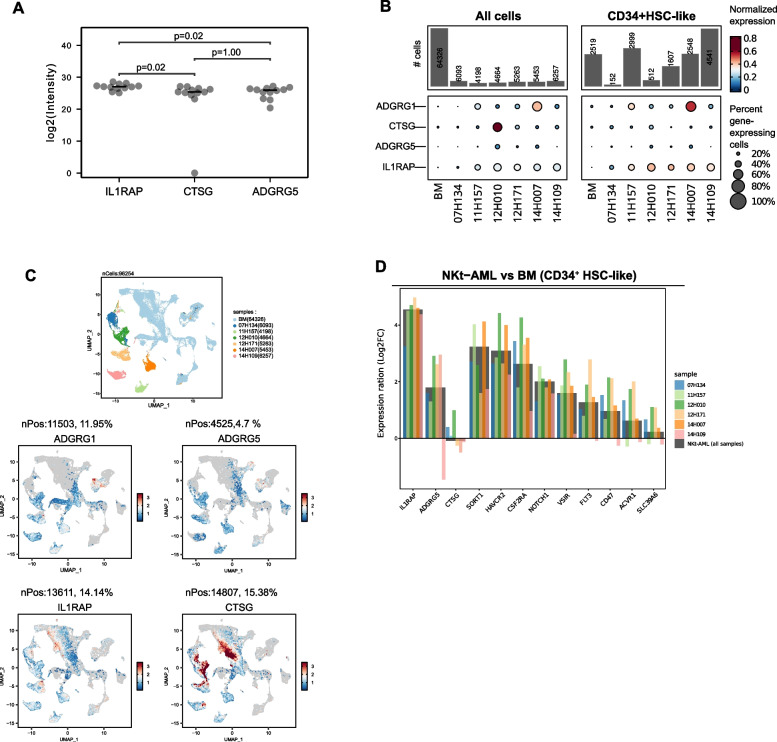
Identification of NKt-AML candidate antigens. **A** Dot plots show candidate antigen protein intensity in NKt-AML samples from the Leucegene AML surfaceome cohort. P values were calculated using the Wilcoxon test. **B** Single cell RNA expression of candidate antigens along with ADGRG1 in all cells (left) and CD34^+^ HSC like cells (right) from NKt-AML samples and normal BM (data from the Human cell atlas). The percentage of gene-expressing cells along with Z-scores calculated per gene for expression in the different samples are shown. Barplots on top represent the number of cells per sample. **C** UMAP representation of single-cell RNA-sequencing data from NKt-AML samples and normal BM colored according to sample identity (top) or to expression of candidate antigens as well as ADGRG1 (bottom). **D** Log2 FC expression ratio of each candidate in CD34 + HSC like cells of NKt-AML vs BM (grey bars) or individual sample vs BM (colored bars, same color code). Antigens in the left panel are selected according to the pipeline. Antigens in the right panel are FDA approved (or clinical Phase I completed) targets, that are selectively expressed on NKt-AML (don’t match selection pipeline)

This analysis provided a list of 3 candidates antigens for NKt-AML with a potentially favorable safety profile, namely CTSG, ADGRG5 and IL1RAP (Fig. 2A). We then validated the expression of these antigens at the transcript level and compared their expression profile in NKt-AML specimens and normal BM samples to identify antigens showing preferential expression by AML cells. To achieve this, we exploited single-cell RNA sequencing data from NKt-AML specimens previously generated by our group (*n* = 3), which were combined with newly generated data (*n* = 3 NKt-AML) and compared to normal BM single-cell RNA-sequencing data from the Human cell atlas [61]. These observations revealed that ADGRG1, strongly overexpressed by NKt-AML at the protein level (Fig. 1C) but not selected in our pipeline based on its strong expression by T cells (data not shown), is expressed asymmetrically among the 6 NKt-AML specimens both in AML cells and in HSC-like AML cells (Fig. 2B-C, umap projection of cell type in Fig. S2 A). CTSG and ADGRG5 were more uniformly but poorly expressed in both AML and HSC-like AML cells (Fig. 2B-C). IL1RAP on the other hand was found to be homogeneously expressed by AML and HSC-like AML cells of all 6 NKt-AML specimens analyzed and showed little expression in BM cells including HSC-like BM cells (Fig. 2B-C and Fig. S2B). Importantly, among the three candidates, IL1RAP was the gene most overexpressed by HSC-like AML cells compared to normal BM HSCs (average log2(FC) = 4.55; Fig. 2D, Fig. S2 C and S2D. Interestingly, among cell surface proteins overexpressed by NKt-AML by surface proteomics (Fig. 1B, *n* = 88) are found several proteins targeted by therapeutic antibodies under clinical development in AML as well as in other cancer and non-cancer indications (Table S2). None of these antigens showed overexpression by NKt-AML HSC-like cells compared to normal BM HSCs by an order of magnitude comparable to IL1RAP however (Fig. 2D). Altogether, these analyses position IL1RAP as the most promising antigen for therapeutic intervention in NKt-AML.

### Assessment of IL1RAP expression profile in AML subgroups

**Fig. 3:**
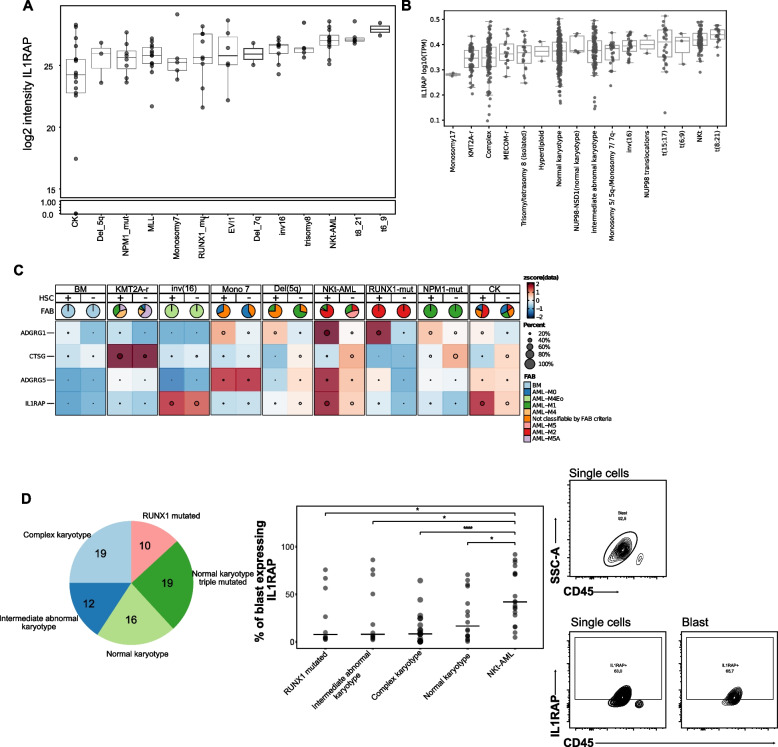
Validation of IL1RAP as a NKt-AML antigen. **A** IL1RAP protein intensity measured by surface proteomics across all AML subgroups of the Leucegene AML surfaceome cohort (*n* = 100). **B** IL1RAP transcript levels measured by RNA sequencing across all AML subgroups of the Leucegene AML RNA-sequencing cohort (*n* = 691). **C** Single-cell RNA expression of candidate antigens and ADGRG1 in normal BM and AML specimens of the Leucegene AML single-cell RNA-sequencing cohort (*n* = 20) in all AML cells (HSC: -) or HSC-like AML cells (HSC: +). The percentage of gene-expressing cells along with Z-scores calculated per gene for expression in the different AML subgroups and normal BM are shown. **D** Validation of IL1RAP cell surface expression in primary human AML specimens by flow cytometry. Left) Distribution of AML subgroups within the cohort of primary human AML specimens analyzed by flow cytometry. Middle) Percentage of IL1RAP-expressing blasts in specimens of indicated AML subgroups. P values were calculated using the Wilcoxon test. Right) Flow cytometry analysis of IL1RAP expression in NKt-AML specimens. Profile from 1 representative specimen is shown

Although IL1RAP has been identified as an antigen for NKt-AML, we wished to determine if IL1RAP is also expressed by other AML subgroups, as one could envision that therapeutic strategies targeting this antigen could be extended to other AML subtypes. Results show that IL1RAP is most highly expressed by t(6;9), t(8;21) and NKt-AML (*n* = 12) both at the protein (surface proteomics, Leucegene AML surfaceome cohort (*n* = 100), Fig. 3A) and transcript levels (transcriptome, Leucegene RNA-sequencing cohort (*n* = 691), Fig. 3B, t(8;21) Fig. S3 A). Complex karyotype AML showed lowest IL1RAP protein intensities with some variation (Fig. 3A, CTSG and ADGRG5 Fig. S3B).

Exploration of IL1RAP expression in AML subtypes was then pursued using single-cell RNA sequencing data generated from 20 specimens of the AML surfaceome cohort (Fig. 3C). These analyses confirmed that NKt-AML expresses high IL1RAP levels (Fig. 3C). Furthermore, ADGRG5 is only expressed in a small percentage of cells, including in NKt-AML and CTSG shows a higher expression in non-HSC-like NKt-AML cells. Interestingly, a marked increase in IL1RAP expression was observed in HSC-like AML cells compared to other AML cell types in NKt-AML (Fig. 3C). Again, normal BM-derived HSCs showed low IL1RAP transcript levels, and IL1RAP expression profile was similar in BM HSCs and other BM cell types (Fig. 3C, first 2 columns, and Fig. S2B). By comparison, ADGRG1 showed a different expression profile than IL1RAP in AML subgroups, and was expressed by more primitive cells both in NKt-AML and normal BM. These results suggest that IL1RAP is preferentially expressed by primitive NKt-AML cells, an observation not recapitulated in normal BM.

To further validate these findings, flow cytometry analyses were conducted on a cohort of 76 randomly selected primary human AML specimens which included 19 NKt-AML, 16 NK, 12 intermediate abnormal karyotype, 19 complex karyotype and 10 RUNX1-mutated AML specimens. Results, presented in Fig. 3D, confirm that NKt-AML expresses IL1RAP in a significantly greater proportion of blasts than the other 4 AML subgroups analyzed.

### FLT3-ITD mutations associate with elevated IL1RAP expression

**Fig. 4:**
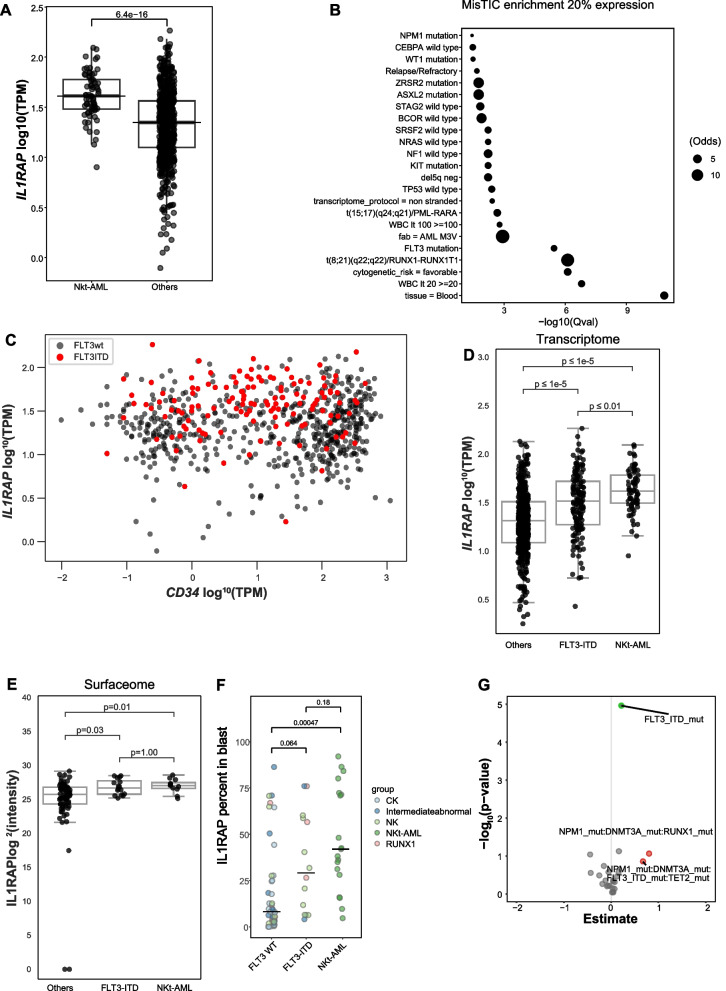
FLT3-ITD mutations associate with elevated IL1RAP expression. **A** IL1RAP transcript levels in NKt-AML and other specimens of the Leucegene AML RNA-sequencing cohort (*n* = 691). P value calculated with Wilcoxon test. **B** Enrichment analysis of specimen characteristics in top 20% specimens with highest IL1RAP levels compared to other specimens of the Leucegene AML RNA-sequencing cohort. **C** Scatter plot of IL1RAP and CD34 (log10 TPM + 1). FLT3-ITD mutated samples are shown in red. **D** Transcriptomic analysis of IL1RAP expression in NKt-AML, FLT3-ITD AML and samples from other AML subgroups of the Leucegene AML RNA-sequencing cohort. P values were calculated using the Wilcoxon test. **E** IL1RAP protein intensity measured by surface proteomics in NKt-AML, FLT3-ITD AML and samples from other AML subgroups of the Leucegene AML surfaceome cohort. P values were calculated using the Wilcoxon test. **F** Percentage of IL1RAP-expressing blasts determined by flow cytometry in NKt-AML, FLT3-ITD and FLT3 WT AML specimens. Bars represent medians. P values were calculated using the Wilcoxon test. **G** Multivariate regression analysis of IL1RAP transcript levels, regarding status of NPM1, DNMT3 A, FLT3-ITD, RUNX1, and TET2 mutation and all mutation combination. P values were calculated using the Wilcoxon test

To extend the investigation of IL1RAP expression profile in AML subgroups and more precisely identify patient characteristics associated with elevated IL1RAP expression, we performed transcriptome studies using the entire Leucegene collection of 691 (table S4) primary human AML specimens that included 69 NKt-AML samples. These analyses show that IL1RAP is uniformly expressed in NKt-AML specimens of the Leucegene cohort and is significantly overexpressed by NKt-AML compared to samples from other AML subgroups of the collection (Fig. 4A, *p* = 6.4e- 16). Transcriptomic data also confirmed that t(8;21) AML uniformly expresses high levels of IL1RAP (Fig. 4B). We next performed enrichment analyzes to identify additional features associated with IL1RAP expression in the Leucegene cohort using the MiSTIC tool developed by our group [69]. These analyses revealed several associations between specimen characteristics and elevated IL1RAP expression (top 20% IL1RAP expressors vs others), with FLT3-ITD representing the mutation most significantly associated with IL1RAP expression (Fig. 4B-C and table S5) and blood sample the most associated tissue. Importantly, NKt-AML samples do not exhibit a clear association with a specific tissue type, as evidenced by the comparable distribution of blood (*n* = 34) and bone marrow (*n* = 35) samples. This observation implies that while blood-sampled tissue may be associated with high IL1RAP expression, the specific tissue type, whether blood or bone marrow, does not appear to be a significant factor in IL1RAP NKt-AML association. In line with these results, FLT3-ITD and NKt-AML showed significantly higher IL1RAP levels than other specimens of the cohort both at transcript and protein levels (Fig. 4D-E). A similar trend was also observed by flow cytometry analysis (Fig. 4F). IL1RAP was also overexpressed by NKt-AML compared to FLT3-ITD AML at the transcript level in the Leucegene cohort, suggesting NPM1 and DNMT3 A mutations might contribute to elevated IL1RAP expression (Fig. 4D). A similar trend was also observed by flow cytometry analysis (Fig. 4F). Multivariate regression analyses indicated that FLT3-ITD mutations represent the most significant predictor of IL1RAP overexpression (Fig. 4G). Altogether, these results demonstrate that FLT3-ITD mutations, either alone or in the context of NKt-AML, strongly associate with elevated IL1RAP expression.

### High IL1RAP expression predicts poor survival and nonresponse to HSCT

**Fig. 5:**
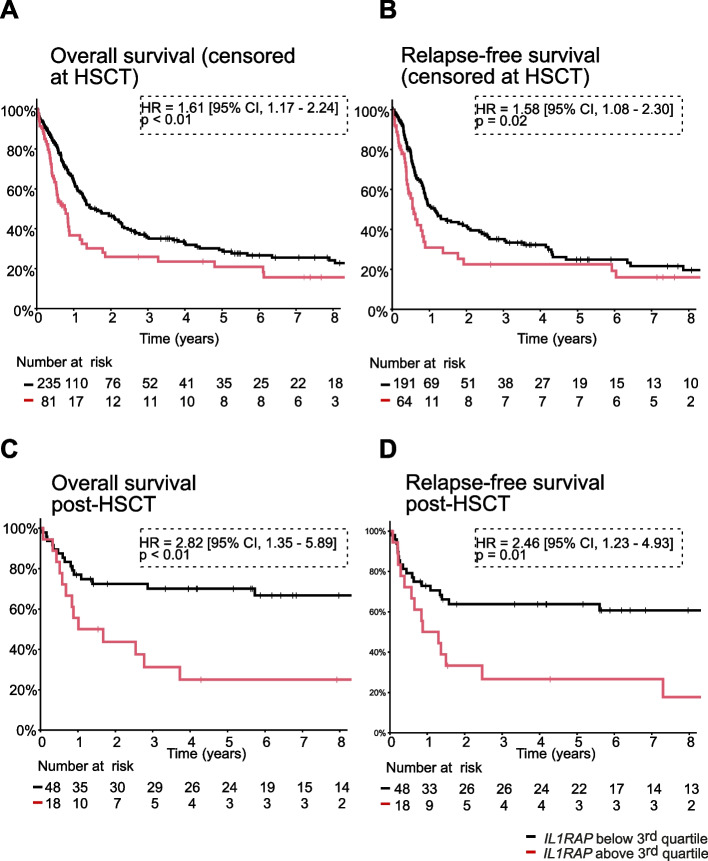
High IL1RAP expression predicts poor survival and nonresponse to HSCT. Overall survival (**A**&**C**) and relapse-free survival (**B**&**D**) censored at HSCT (**A**&**B**) or post-HSCT (**C**&**D**) of intermediate-risk AML patients of the Leucegene cohort (*n* = 316, annotated according to the 2022 ELN classification) according to IL1RAP transcript levels determined by RNA sequencing

The association between IL1RAP expression and AML patient survival and response to hematopoietic stem cell transplantation (HSCT) was next examined, as FLT3-ITD mutations have been reported to associate with decreased overall survival in NPM1-mutated/DNMT3 A-mutated AML [8]. For this purpose, we focused on the intermediate-risk AML subset of the Leucegene cohort of AML patients (*n* = 316, annotated according to the 2022 ELN classification [1]). This cohort of patients was dissociated in two groups based on IL1RAP expression and we observed that patients with IL1RAP expression levels above the 3rd quartile display worst overall survival (*p* < 0.01, HR = 1.61 [95% CI, 1.17—2.24]) and relapse-free survival (*p* = 0.02, HR = 1.58 [95% CI, 1.08—2.30]), censored at HSCT, than patients with IL1RAP expression below the 3rd quartile (Fig. 5A-B). The impact of IL1RAP expression at diagnosis was most striking for patients that underwent allogenic HSCT as consolidation therapy. Indeed, for this subgroup of 66 patients, high IL1RAP expression strongly associated with reduced overall survival (*p* < 0.01, HR = 2.82 [95% CI, 1.35–5.89]) and relapse-free survival (*p* = 0.01, HR = 2.46 [95% CI, 1.23–4.93], Fig. 5C-D). These results importantly highlight an unmet medical need, that is the urgency to identify alternative therapeutic strategies for AML patients with elevated IL1RAP expression, as these patients do not appear to benefit from standard chemotherapy or HSCT.

### IL1RAP as a potential therapeutic target

**Fig. 6:**
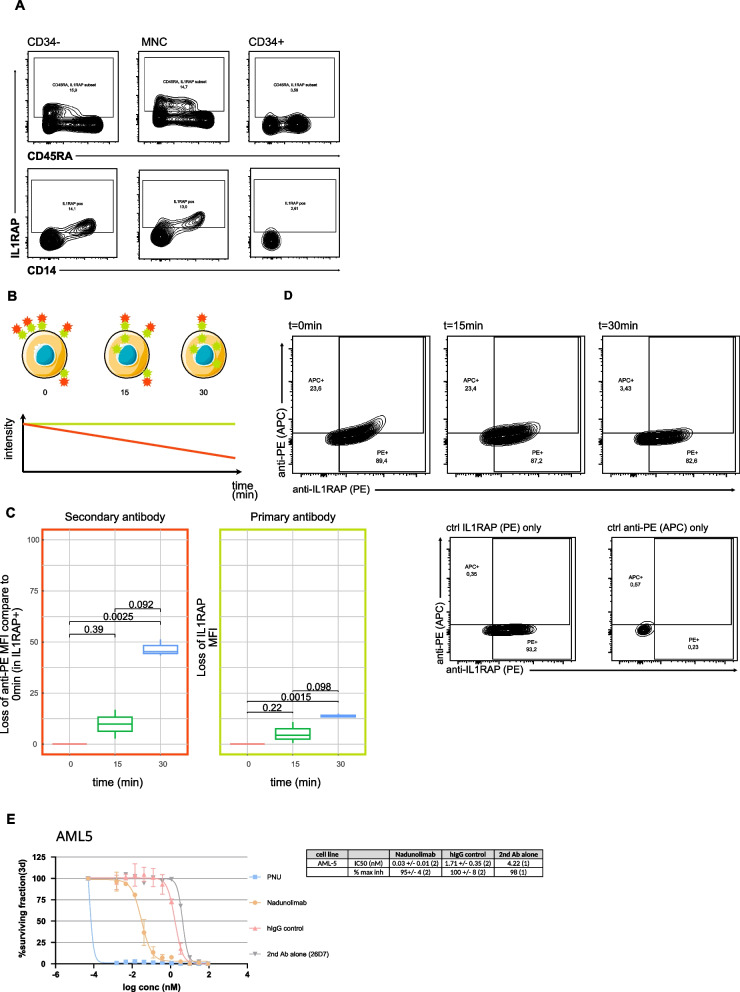
IL1RAP as therapeutic target. **A** IL1RAP expression in BM CD34-, MNC, CD34 + sorted population. **B** Schematic representation of internalization assay. OCI-AML3 cells were stained with PE-conjugated IL1RAP antibodies at t = 0 min and incubated at 37 °C for indicated times. At t = 15 and 30 min, cells are stained with APC-conjugated anti-PE antibodies. Upon IL1RAP and PE-conjugated IL1RAP antibody internalization, APC signal should decrease with time while PE signal should remain constant. **C** Flow cytometry analysis of OCI-AML3 cells at different time points during internalization assay. P values were calculated using the Wilcoxon test. **D** Flow cytometry profile of anti-IL1RAP and anti-PE at different time point. **E** Growth inhibition assay with AML5 treated with Nadunolimab with PNU-conjugated secondary antibody (*n* = 2), PNU (*n* = 2), IgG contro l(*n* = 2), secondary antibody only (*n* = 1)

As previously mentioned, IL1RAP was selected as a NKt-AML antigen based on its potentially favorable safety profile due to its limited expression in normal essential organ tissues and hematopoietic cell populations (Fig. S2). Provided that IL1RAP expression in normal hematopoietic cells was mainly evaluated at the transcript level in contrast to normal organ tissues, we wished to validate these findings via flow cytometry analysis. Transcriptome and single-cell RNA sequencing analyses revealed preferential IL1RAP expression by committed hematopoietic cell types such as granulocytes and neutrophils from normal peripheral blood and BM samples (Fig. S2B), limited expression by HSC-enriched cell populations from umbilical cord blood and normal BM, and extremely low to undetectable levels in platelets [43, 60] and T cells (Fig. 6A and Fig. S2B and S1). Flow cytometry analysis of normal BM showed that approximately 15% of BM cells express IL1RAP, with IL1RAP expression mostly limited to the CD34- fraction (Fig. 6A), consistent with transcriptomic and single cell data showing IL1RAP expression in normal hematopoietic cells (Fig. 6A and Fig. S1). Only a minor subset of CD34 + cells expressed IL1RAP at their surface and CD34 + CD45RA- HSC-enriched populations expressed very low levels of IL1RAP (Fig. 6A), as observed by transcriptomics (Figs. S1B). Further dissection of the IL1RAP + population revealed that this fraction co-expresses high levels of CD14, consistent with transcriptomic data showing highest IL1RAP expression in monocyte. Together these results suggest that IL1RAP ablation may not provoke undue toxicity in humans as primitive normal hematopoietic cells, platelet and T cells express low to undetectable levels of IL1RAP.

To establish IL1RAP as an actionable biological target for NKt-AML cell elimination, we tested the internalization potential of IL1RAP antibodies upon IL1RAP binding at the surface of AML cells, which is a pre-requisite for drug release by antibody–drug conjugates (ADCs). To achieve this, OCI-AML3 cells were first incubated with anti-IL1RAP PE-conjugated primary antibodies and then stained with anti-PE APC-conjugated secondary antibodies at various time points (Fig. 6B). In the case of IL1RAP antibody internalization, secondary antibody signal (APC) is expected to decrease with time while primary antibody signal (PE) should remain relatively constant (Fig. 6B). Results show 50% reduction of secondary antibody signal after 30 min, as opposed to 17% reduction of primary antibody signal (Fig. 6C-D). These experiments demonstrate that IL1RAP antibody is internalized upon IL1RAP binding on AML cells, positioning IL1RAP as a promising ADC target in AML. Next, we explored the possibility of eradicating the AML5 cell line by employing Antibody Drug Conjugate (ADC). This approach utilized Nadunolimab (developed by Cantargia, NCT06548230) as the primary antibody and PNU-conjugated anti-hFc antibodies as the secondary antibodies. The results revealed that the combination of Nadunolimab and the PNU-conjugated secondary antibody was effective in targeting and eliminating the AML5 cell line. This potent effect was observed at a notably low IC50 value of 0.041 nM. In contrast, when the PNU-conjugated secondary antibody was used alone, it exhibited a significantly higher IC50 value of 4.22 nM, indicating a substantially weaker cytotoxic effect (Fig. 6E). An IL1RAP-negative control cell line was also used (U266), and it demonstrated no response to Nadunolimab with PNU-conjugated secondary antibody, with an IC50 value of 46.45 nM (Fig. S4).

These results strongly suggest that targeting IL1RAP using ADC, could potentially serve as a highly effective therapeutic strategy for targeting and eradicating NKt-AML.

## Discussion

NKt-AML is a disease that confers poor prognosis to afflicted patients and hence represents an unmet medical need. Additionally, the poor prognosis associated with NKt-AML continues to pose a significant challenge to hematologists as NKt-AML is associated with a significantly lower sensitivity to daunorubicin, commonly used in combination with cytarabine in the 7 + 3 protocol [70]. Using surface proteomics and single-cell transcriptomics, we identified IL1RAP as the most promising antigen for immunotherapeutic targeting in AML. Importantly, we demonstrated that primitive AML cells significantly overexpress IL1RAP compared to normal BM-derived HSCs, suggesting that IL1RAP targeting could potentially spare normal BM HSCs. We show that IL1RAP expression associates with FLT3-ITD mutation occurrence and might be enhanced by NPM1 and DNMT3 A mutations, hence its predominance in NKt-AML. Furthermore, we show that IL1RAP is internalized in leukemic cells upon treatment with IL1RAP antibodies, paving the way for therapeutic intervention with antibody–drug conjugates. A recent paper by Zhang et al. describes a new IL1RAP-specific T cell engager that depletes primitive leukemic cells, further supporting the potential for IL1RAP as a relevant AML target [29]. Our work nicely complements this paper as it highlights a key AML subgroup in which such therapy may be most effective. Likewise, the studies by Deschamps and Ferrand and collaborators on CAR-T targeting [71] of IL1RAP will benefit from companion testing described herein.

Using single cell RNA sequencing data from a panel of 20 primary AML specimens of various genetic/cytogenetic subgroups, we could further determine that IL1RAP may represent a good therapeutic target in other AML subgroups including t(6;9) AML (only 2 specimens tested) and those with core binding factor translocations.

IL1RAP overexpression has been described in hematological cancers such as CML and AML, as well as in solid tumors such as pancreatic ductal adenocarcinoma, Ewing sarcoma [51], glioma, triple negative breast cancer, non-small cell lung cancer, stomach adenocarcinoma, or cervical cancer [50]. In AML cells, IL1RAP physically interacts with c-kit and FLT3, inducing FLT3 phosphorylation and maintaining cellular proliferation, making it an attractive target for chemotherapeutic intervention. Indeed, IL1RAP knockdown in primary AML cells is accompanied by a reduction in clonogenicity, and BM cells from IL1RAP knockout (IL1RAP-/-) mice transduced with MLL-AF9 fusion viruses show slower disease progression than recipients of wild type cells transduced with the same onco-vector [54]. While IL1RAP-blocking antibodies demonstrated antiproliferative activity, their function is overcome in the presence of FLT3-ITD mutations where FLT3 is constitutionally active (phosphorylated) [50]. These results argue that an IL1RAP blocking antibody might not achieve efficient AML cell elimination in the context of NKt-AML as, by definition, these leukemia harbor FLT3-ITD mutations. Hence our efforts to demonstrate the feasibility of targeting IL1RAP-expressing AML cells with antibody–drug conjugates, a mechanism solely dependent on IL1RAP expression and not expected to be influenced by FLT3 status.

Although we demonstrate that IL1RAP is preferentially expressed by NKt-AML, our results indicate that other AML subgroups such as t(6;9) and t(8;21) AML express relatively high levels of IL1RAP and might benefit from immunotherapeutic strategies targeting IL1RAP. Considering that different IL1RAP-targeting agents have been developed for several cancer indications (for example Nadunolimab, a monoclonal IL1RAP antibody exhibiting antibody-dependent cellular cytotoxicity and receptor blocking activities, as well as CAR-T cells targeting IL1RAP under early clinical development [71–73]), our work will help position these novel therapies in AML by identifying AML patients most likely to respond to IL1RAP-targeting immunotherapeutic strategies and possibly provide logical grounds for combinatorial treatments with existing agents.

Despite the significant findings of this study, several limitations should be acknowledged. Our surface proteome analysis was conducted on 100 primary AML specimens, including only 12 NKt-AML samples. While this represents a substantial achievement, a larger cohort might provide additional insights into the heterogeneity of antigen expression across and within AML subgroups and a better statistic power. Additionally, flow cytometry validation, while robust for NKt-AML, did not comprehensively cover all cytogenetic subgroups that showed high IL1RAP expression in our omics analyses, particularly t(8;21) and t(6;9) AML. Finally, in vivo ADC targeting assay in patient-derived xenograft models would be valuable to fully assess efficacy and potential off-target effects prior to clinical translation. Nevertheless, the integration of multiple omics approaches and flow cytometry validation provides a robust foundation for identifying IL1RAP as a promising target in NKt-AML with potential broader applications in other AML subtypes.

## Conclusion

Through multi-omic analyses combining surface proteomics, bulk RNA sequencing, and single-cell transcriptomics, we establish that IL1RAP is preferentially expressed in NKt-AML cells, particularly in the primitive stem cell-like populations, while showing limited expression in normal bone marrow HSCs. This differential expression pattern suggests a potentially favorable therapeutic window for targeting IL1RAP in NKt-AML. Our findings reveal that elevated IL1RAP expression strongly correlates with FLT3-ITD mutations and predicts poor clinical outcomes, including reduced overall and relapse-free survival in intermediate-risk AML patients. Notably, high IL1RAP expression identifies patients who respond poorly to hematopoietic stem cell transplantation, highlighting a need for alternative therapeutic strategies and/or post-transplant consolidation therapies (e.g. IL1RAP antibody) for this patient population. Antigen internalization upon IL1RAP targeting suggests that ADC-based targeting is potentially feasible. Beyond NKt-AML, our analysis indicates that other AML subtypes, particularly t(6;9) and t(8;21) AML, also express significant levels of IL1RAP, suggesting potential broader applications for IL1RAP-targeted therapies. This work provides a rationale for the clinical development of IL1RAP-directed therapies, particularly for patients with NKt-AML who currently face limited therapeutic options and poor outcomes with standard treatments.

## Supplementary Information

Supplementary Material 1.

## Data Availability

Surface proteome data have been deposited to the ProteomeXchange Consortium via PRIDE partner with the dataset identifier PXD043772, PXD044480 and 10.6019/PXD044480. Single cell RNA-seq data have been deposited int the GEO repository GSE241989. Leucegene transcriptome sequencing from AML specimens (*n*= 691) have been deposited in the GEO repository GSE232130. Surface proteome, single cell RNA-seq and transcritpome are also available at: https://lasa.leucegene.ca/lasa_v2 (usager: reviewer, mot de passe: EzlK3ww6).

## Abbreviations

ADC: Antibody–drug conjugate
ADCC: Antibody-dependent cell cytotoxicity
AML: Acute myeloid leukemia
APC: Allophycocyanin
BiTE: Bispecific T-cell engager
BM: Bone marrow
CAR-T cells: Chimeric antigen receptor T cells
CK: Complex karyotype
CML: Chronic myeloid leukemia
CTSG: Cathepsin G
DNA: Deoxyribonucleic acid
DNMT3A: DNA methyltransferase 3 A
ELN: European Leukemia Network
FACS: Fluorescence-activated cell sorting
FDA: U.S. Food and Drug Administration
FLT3: FMS-like tyrosine kinase 3
FLT3-ITD: FLT3 internal tandem duplication
FSC: Forward scatter
GEO: Gene Expression Omnibus
GRP56: G protein-coupled receptor 56
HCA: Human Cell Atlas
HSC: Hematopoietic stem cell
HSCT: Hematopoietic stem cell transplantation
IL1RAP: Interleukin- 1 receptor accessory protein
ITD: Internal tandem duplication
KMT2 A-r: KMT2 A rearranged
LASA: Leucegene AML surfaceome atlas
LSC: Leukemic stem cell
MECOM-r: MECOM rearranged
MiSTIC: Minimum Spanning Trees Inferred Clustering
MLL: Mixed-lineage leukemia
MNC: Mononuclear cell
mRNA: Messenger RNA
NK: Normal karyotype
NKt-AML: NK triple mutated AML
NPM1: Nucleophosmin 1
NPM1c: Cytoplasmic nucleophosmin 1
NUP98-NSD1: NUP98-NSD1 translocation
OXPHOS: Oxidative phosphorylation
PCA: Principal component analysis
PBS: Phosphate-buffered saline
PBX3: Pre-B-cell leukemia homeobox 3
PE: Phycoerythrin
PRIDE: Proteomics Identification Database
RNA: Ribonucleic acid
rRNA: Ribosomal RNA
RUNX1: RUNX family transcription factor 1
scRNA-seq: Single-cell RNA sequencing
SCP: Single-cell pipeline package
SPAT: Surface protein annotation tool
SSC: Side scatter
TPM: Transcripts per million
UMAP: Uniform Manifold Approximation and Projection
WHO: World Health Organization
WT: Wild type
