# Supplementary material for: IL1RAP is an immunotherapeutic target for normal karyotype triple-mutated acute myeloid leukemia
Source: Biomark Res. 2025 Apr 14;13:61. doi: 10.1186/s40364-025-00769-z (PMC11995633; doi:10.1186/s40364-025-00769-z)
Supplement: Supplementary file 1 — Supplementary Material 1. [file 40364_2025_769_MOESM1_ESM.pdf]

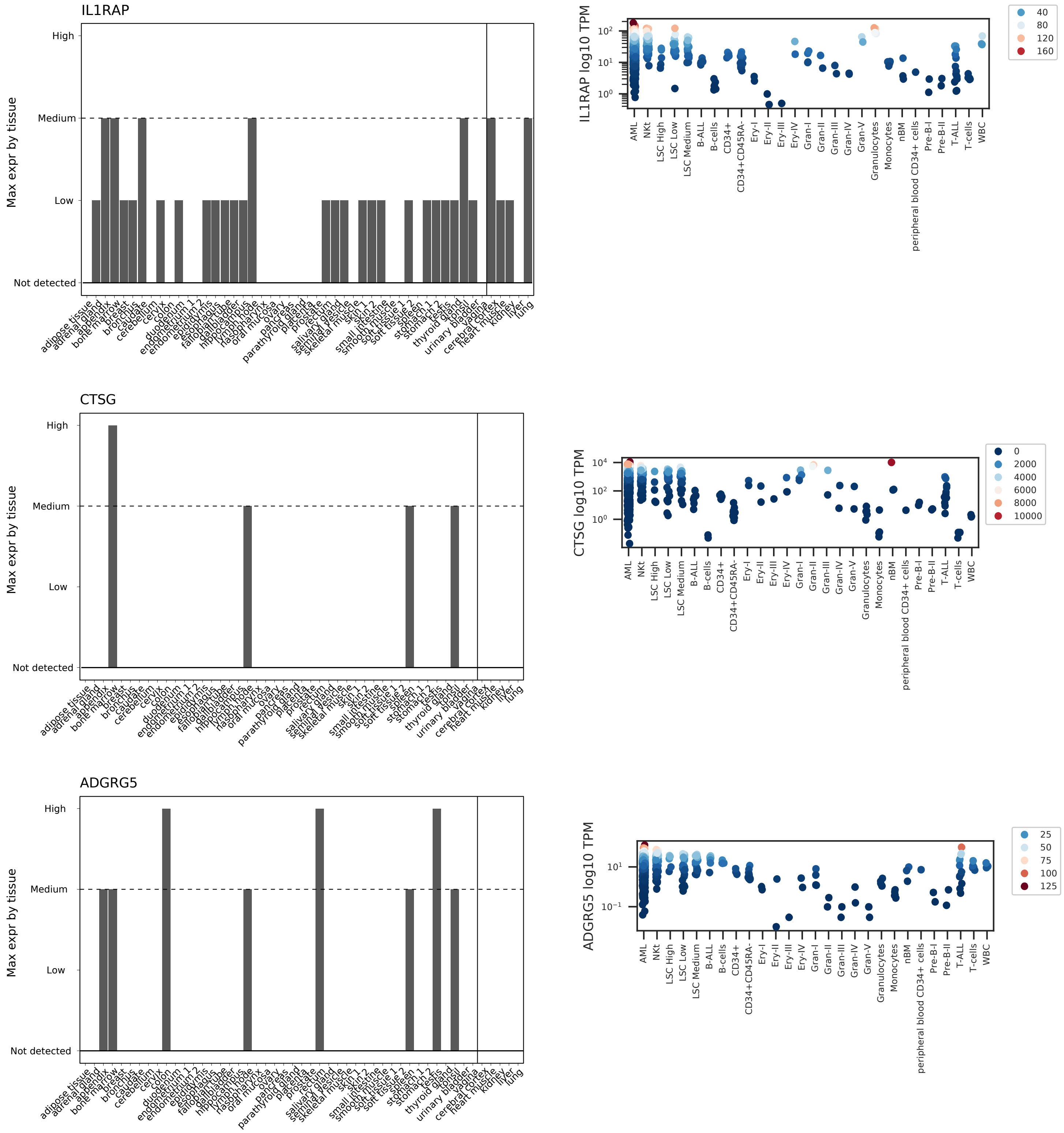

Figure S1: Human protein atlas profile of IL1RAP, ADGRG5 and CTSG and human hematopoietic populations.

Right panel : Essential organ (cerebral cortex, heart muscle, kidney, lung) are plotted at the righ of the verticalseparator. Left panel : Expression in normal human hematopoietic population and AML

A

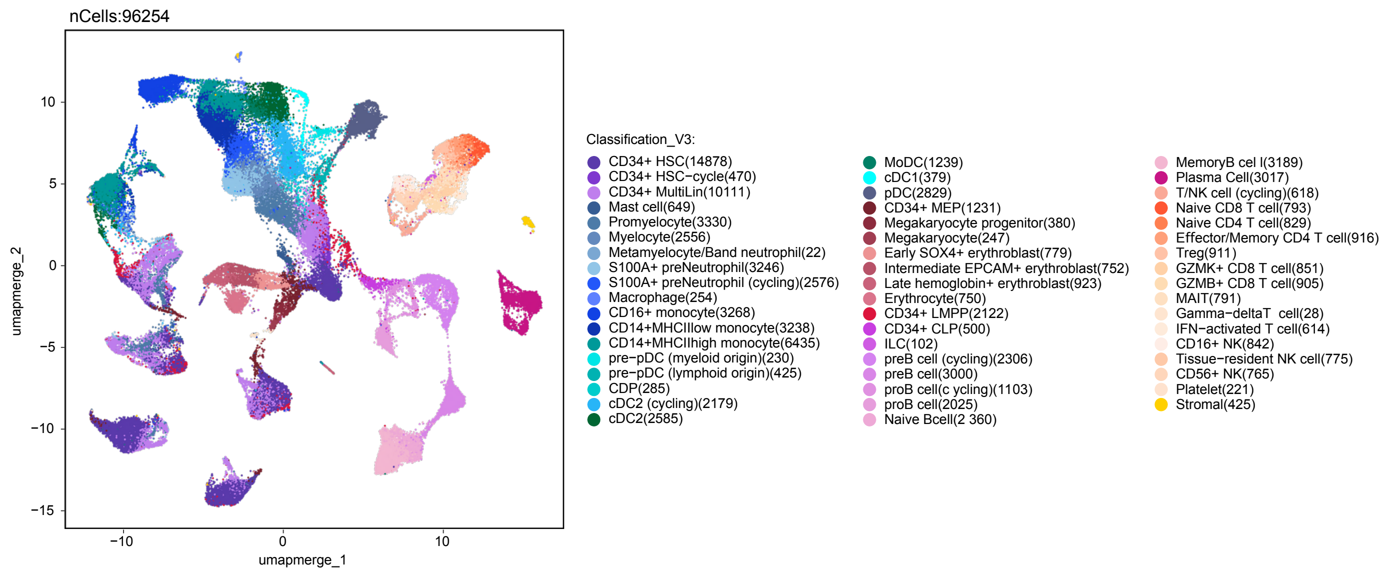

B

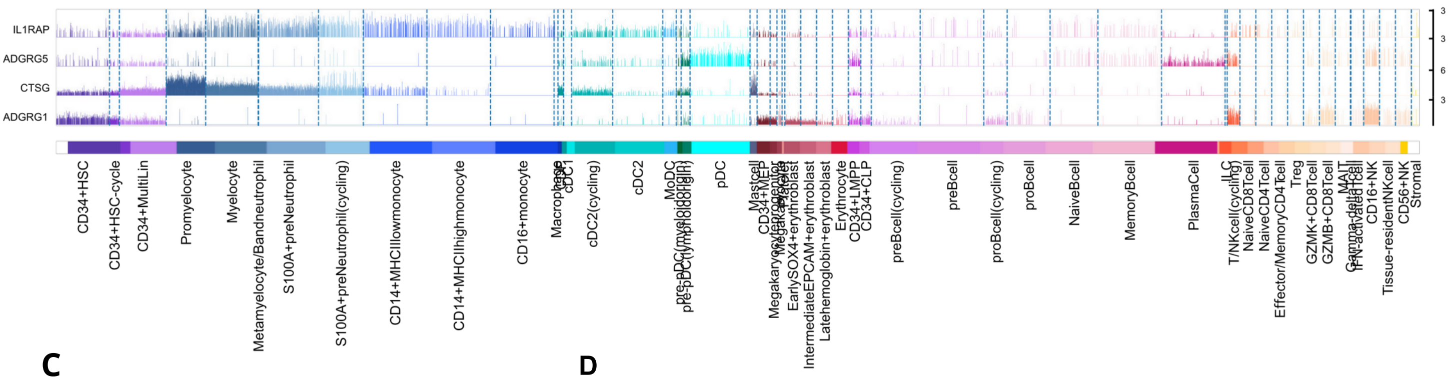

C

p_val	avg_log2FC	pct.1	pct.2	p_val_adj	gene
0.00e+00	4.55e+00	0.429	0.016	0.00e+00	IL1RAP
1.63e-180	1.59e+00	0.596	0.279	5.96e-176	VSIR
6.89e-145	2.18e+00	0.345	0.080	2.52e-140	IL3RA
7.13e-95	1.27e+00	0.435	0.197	2.61e-90	FLT3
1.36e-89	9.58e-01	0.637	0.416	4.99e-85	CD47
1.83e-74	3.24e+00	0.144	0.012	6.72e-70	SORT1
2.90e-69	2.01e+00	0.182	0.039	1.06e-64	NOTCH1
1.81e-52	2.62e+00	0.118	0.017	6.62e-48	CSF2RA
2.41e-35	3.09e+00	0.072	0.007	8.83e-31	HAVCR2
5.28e-28	1.79e+00	0.078	0.017	1.93e-23	ADGRG5
4.15e-08	2.35e-01	0.152	0.103	1.52e-03	SLC39A6
2.67e-06	6.26e-01	0.045	0.024	9.77e-02	ACVR1
4.97e-01	-8.51e-02	0.138	0.127	1.00e+00	CTSG

D

p_val	avg_log2FC	pct.1	pct.2	p_val_adj	sample	gene
3.92e-60	3.25e+00	0.250	0.016	1.43e-55	07H134	IL1RAP
3.88e-47	3.43e+00	0.217	0.017	1.42e-42	07H134	CSF2RA
5.91e-42	2.32e+00	0.428	0.080	2.17e-37	07H134	IL3RA
3.43e-33	1.53e+00	0.842	0.416	1.25e-28	07H134	CD47
1.18e-20	1.54e+00	0.625	0.279	4.33e-16	07H134	VSIR
6.13e-19	2.71e+00	0.112	0.012	2.25e-14	07H134	SORT1
4.26e-15	1.03e+00	0.500	0.197	1.56e-10	07H134	FLT3
5.71e-10	2.64e+00	0.059	0.007	2.09e-05	07H134	HAVCR2
1.75e-08	1.60e+00	0.086	0.017	6.39e-04	07H134	ADGRG5
1.30e-06	1.31e+00	0.125	0.039	4.76e-02	07H134	NOTCH1
1.99e-05	6.71e-01	0.230	0.103	7.28e-01	07H134	SLC39A6
1.85e-03	1.35e+00	0.066	0.024	1.00e+00	07H134	ACVR1
1.51e-02	3.94e-01	0.204	0.127	1.00e+00	07H134	CTSG
1.32e-229	4.51e+00	0.377	0.016	4.83e-225	11H157	IL1RAP
8.67e-211	1.87e+00	0.664	0.279	3.18e-206	11H157	VSIR
2.56e-104	4.02e+00	0.199	0.012	9.39e-100	11H157	SORT1
2.59e-79	2.54e+00	0.212	0.039	9.49e-75	11H157	NOTCH1
1.44e-74	1.88e+00	0.271	0.080	5.26e-70	11H157	IL3RA
1.33e-45	6.82e-01	0.594	0.416	4.87e-41	11H157	CD47
1.09e-37	7.99e-01	0.361	0.197	3.98e-33	11H157	FLT3
1.49e-18	2.86e+00	0.047	0.007	5.46e-14	11H157	HAVCR2
9.76e-16	1.79e+00	0.059	0.017	3.57e-11	11H157	CSF2RA
6.67e-11	1.29e+00	0.049	0.017	2.44e-06	11H157	ADGRG5
8.68e-02	8.25e-02	0.145	0.127	1.00e+00	11H157	CTSG
2.61e-01	-1.96e-01	0.095	0.103	1.00e+00	11H157	SLC39A6
5.27e-01	-2.87e-01	0.021	0.024	1.00e+00	11H157	ACVR1
9.06e-233	4.70e+00	0.480	0.016	3.32e-228	12H010	IL1RAP
3.15e-198	3.58e+00	0.615	0.080	1.15e-193	12H010	IL3RA
3.76e-167	2.79e+00	0.818	0.279	1.38e-162	12H010	VSIR
1.13e-151	2.15e+00	0.881	0.416	4.14e-147	12H010	CD47
4.10e-126	4.27e+00	0.303	0.017	1.50e-121	12H010	CSF2RA
9.72e-90	1.90e+00	0.629	0.197	3.56e-85	12H010	FLT3
4.14e-77	4.42e+00	0.174	0.007	1.51e-72	12H010	HAVCR2
1.09e-56	2.91e+00	0.176	0.017	3.99e-52	12H010	ADGRG5
1.33e-34	2.10e+00	0.188	0.039	4.86e-30	12H010	NOTCH1
2.98e-28	2.58e+00	0.100	0.012	1.09e-23	12H010	SORT1
4.70e-18	1.10e+00	0.254	0.103	1.72e-13	12H010	SLC39A6
8.75e-16	1.74e+00	0.098	0.024	3.20e-11	12H010	ACVR1
2.31e-11	9.89e-01	0.250	0.127	8.45e-07	12H010	CTSG

p_val	avg_log2FC	pct.1	pct.2	p_val_adj	sample	gene
8.88e-235	2.78e+00	0.650	0.197	3.25e-230	12H171	FLT3
1.74e-228	2.12e+00	0.765	0.416	6.37e-224	12H171	CD47
8.52e-206	4.97e+00	0.368	0.016	3.12e-201	12H171	IL1RAP
3.77e-183	2.33e+00	0.647	0.279	1.38e-178	12H171	VSIR
4.29e-86	2.62e+00	0.305	0.080	1.57e-81	12H171	IL3RA
1.12e-48	3.30e+00	0.127	0.017	4.09e-44	12H171	CSF2RA
1.47e-29	2.61e+00	0.093	0.017	5.39e-25	12H171	ADGRG5
1.42e-17	1.80e+00	0.105	0.039	5.19e-13	12H171	NOTCH1
2.26e-16	2.00e+00	0.078	0.024	8.26e-12	12H171	ACVR1
2.11e-11	1.08e+00	0.171	0.103	7.71e-07	12H171	SLC39A6
1.47e-10	-2.61e-01	0.063	0.127	5.40e-06	12H171	CTSG
3.07e-09	2.64e+00	0.031	0.007	1.13e-04	12H171	HAVCR2
9.33e-08	1.60e+00	0.037	0.012	3.42e-03	12H171	SORT1
1.15e-294	4.61e+00	0.464	0.016	4.20e-290	14H007	IL1RAP
1.88e-287	2.88e+00	0.575	0.080	6.87e-283	14H007	IL3RA
1.07e-260	1.84e+00	0.772	0.279	3.93e-256	14H007	VSIR
1.23e-163	1.17e+00	0.798	0.416	4.51e-159	14H007	CD47
1.15e-161	4.12e+00	0.288	0.012	4.20e-157	14H007	SORT1
5.44e-148	1.46e+00	0.585	0.197	1.99e-143	14H007	FLT3
7.45e-132	3.54e+00	0.255	0.017	2.73e-127	14H007	CSF2RA
4.58e-94	2.96e+00	0.199	0.017	1.68e-89	14H007	ADGRG5
1.50e-80	4.00e+00	0.153	0.007	5.50e-76	14H007	HAVCR2
1.41e-79	2.08e+00	0.223	0.039	5.16e-75	14H007	NOTCH1
5.65e-18	3.75e-01	0.204	0.103	2.07e-13	14H007	SLC39A6
2.71e-10	6.87e-01	0.060	0.024	9.91e-06	14H007	ACVR1
7.27e-01	-5.03e-01	0.133	0.127	1.00e+00	14H007	CTSG
0.00e+00	4.37e+00	0.464	0.016	0.00e+00	14H109	IL1RAP
2.25e-56	1.03e+00	0.246	0.080	8.24e-52	14H109	IL3RA
8.36e-53	1.58e+00	0.168	0.039	3.06e-48	14H109	NOTCH1
3.21e-26	1.74e+00	0.070	0.012	1.18e-21	14H109	SORT1
1.14e-19	2.25e+00	0.048	0.007	4.17e-15	14H109	HAVCR2
1.07e-12	9.65e-01	0.052	0.017	3.92e-08	14H109	CSF2RA
5.17e-12	1.57e-01	0.409	0.279	1.89e-07	14H109	VSIR
4.69e-10	-9.27e-02	0.300	0.197	1.72e-05	14H109	FLT3
1.45e-03	-2.17e-01	0.139	0.103	1.00e+00	14H109	SLC39A6
4.89e-02	-1.36e-01	0.033	0.024	1.00e+00	14H109	ACVR1
5.41e-02	-1.46e+00	0.012	0.017	1.00e+00	14H109	ADGRG5
1.71e-01	-1.31e-01	0.148	0.127	1.00e+00	14H109	CTSG
6.12e-01	-2.60e-01	0.495	0.416	1.00e+00	14H109	CD47

Figure S2: IL1RAP single cell expression in normal hematopoietic tissue.

A) Umap projection with cell type annotation.

B) Track plot showing expression of IL1RAP, ADGRG5, CTSG, and ADGRG1 in normal BM

C) Single cell differential expression of potential target in NKt-AML (HSC-like) vs BM (HSC).

D) Single cell differential expression of potential target in NKt-AML (HSC-like) vs BM (HSC) for each sample individually.

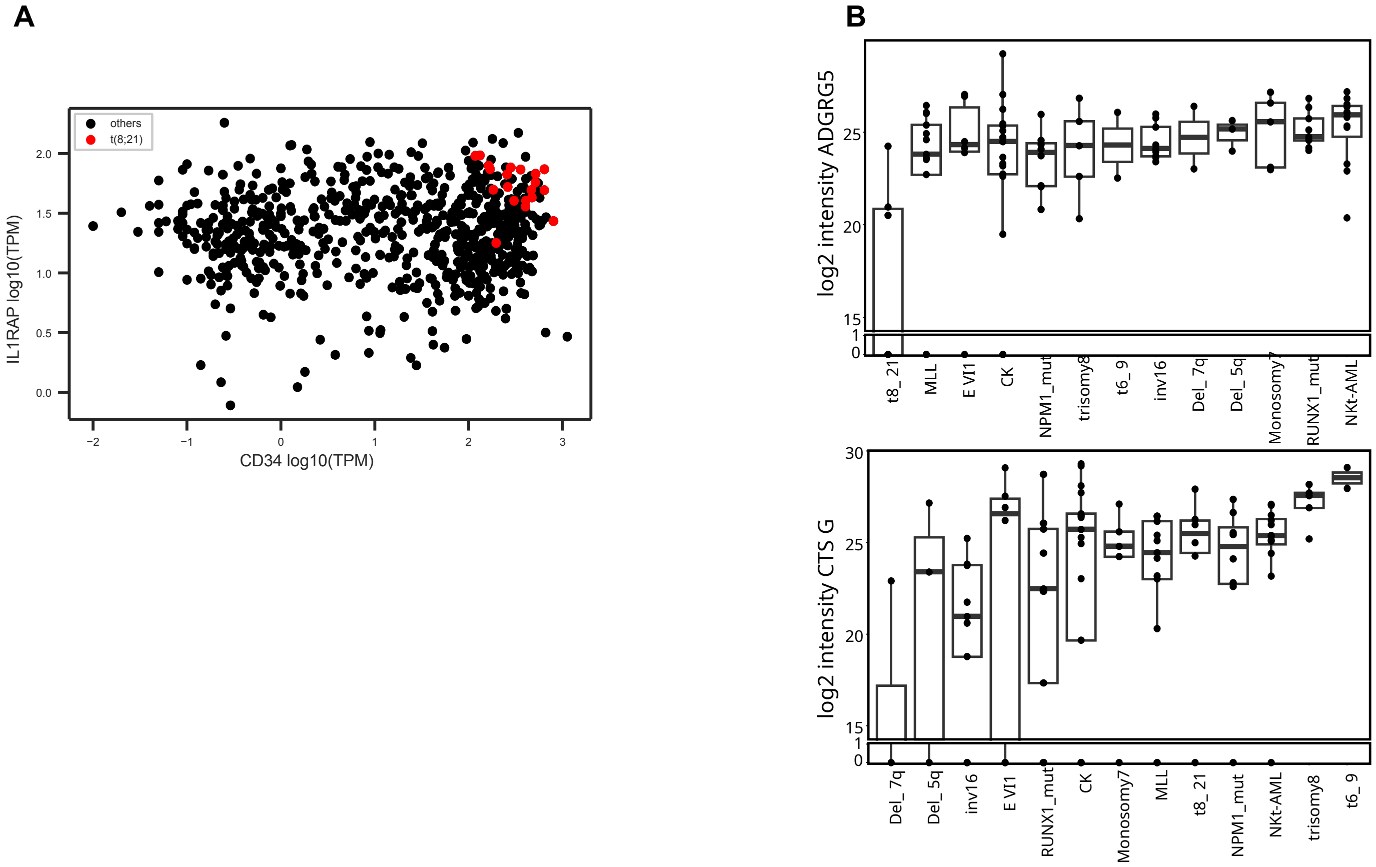

Figure S3: IL1RAP is expressed in others subgroup.

A) Scatter plot showing that t(8;21) AML are enriched in IL1RAP high CD34 high.

B) ADGRG5 and CTSG Surfaceome detection intensity by subrgroup.

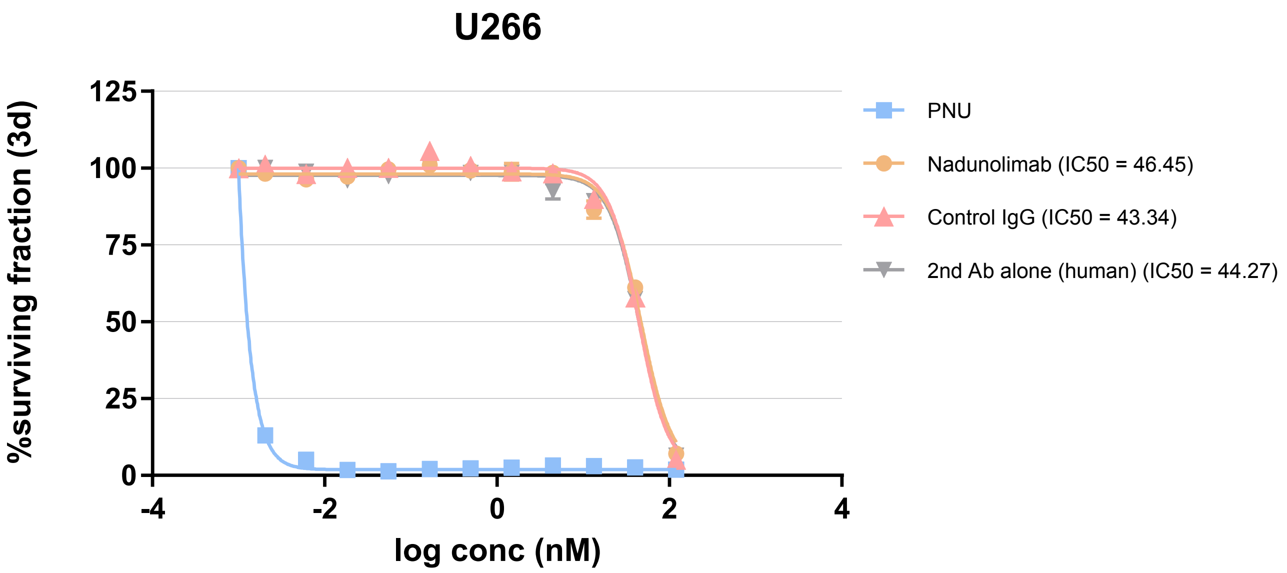

Figure S4 : Indirect inhibition assay on U266 IL1RAP negative control cell line.
Growth inhibition assay with U266 cell line treated with Nadunolimab with PNU-conjugated secondary antibody (n=3), PNU (n=3), IgG control (n=3), secondary antibody only (n=2). All replicates are technical replicates.

sample	Cytogenetic group	mutations	FAB	Age_at_diagnosis	Sex	Nkt-AML
03H030	Complex karyotype	-	AML-M0	82	F	Others
03H090	Complex karyotype	-	AML-M1	63	F	Others
04H132	Complex karyotype	-	AML-M4	46	M	Others
06H029	Complex karyotype	-	AML-M1	20	F	Others
06H074	Complex karyotype	-	AML-M5A	67	M	Others
06H135	Complex karyotype	-	AML-M0	65	M	Others
07H038	Complex karyotype	-	AML-M0	66	M	Others
10H014	Complex karyotype	-	AML-M0	77	M	Others
10H130	Complex karyotype	-	Not classifiable by FAB criteria	56	M	Others
11H103	Complex karyotype	-	AML-M1	65	M	Others
11H170	Complex karyotype	-	AML-M0	51	F	Others
12H106	Complex karyotype	-	Not classifiable by FAB criteria	67	M	Others
12H138	Complex karyotype	-	AML-M2	81	F	Others
12H170	Complex karyotype	-	Not classifiable by FAB criteria	60	F	Others
13H018	Complex karyotype	-	Not classifiable by FAB criteria	69	F	Others
18H027	Complex karyotype	-	AML-M1	57	M	Others
18H047	Complex karyotype	-	AML-M0	60	M	Others
05H034	Deletion 5q (not complex)	-	AML-M1	47	M	Others
11H097	Deletion 5q (not complex)	-	AML-M1	68	F	Others
17H065	Deletion 5q (not complex)	-	Not classifiable by FAB criteria	63	F	Others
10H107	MECOM rearranged	-	AML-M1	31	F	Others
11H205	MECOM rearranged	-	Not classifiable by FAB criteria	83	M	Others
14H038	MECOM rearranged	-	Not classifiable by FAB criteria	49	M	Others
17H105	MECOM rearranged	-	AML-M0	45	M	Others
17H154	MECOM rearranged	-	Not classifiable by FAB criteria	59	M	Others
18H094	MECOM rearranged	-	AML-M2	64	M	Others
03H109	inv(16)	-	AML-M4Eo	29	M	Others
03H112	inv(16)	-	AML-M4Eo	36	M	Others
04H061	inv(16)	-	AML-M4Eo	69	F	Others
05H113	inv(16)	-	AML-M4Eo	63	F	Others
06H151	inv(16)	-	AML-M1	38	F	Others
07H099	inv(16)	-	Not classifiable by FAB criteria	58	F	Others
07H144	inv(16)	-	AML-M4Eo	53	M	Others
09H016	inv(16)	-	AML-M4Eo	64	F	Others
12H042	inv(16)	-	AML-M4Eo	58	M	Others
02H017	KMT2A rearranged	-	AML-M0	50	F	Others
04H121	KMT2A rearranged	-	AML-M5A	32	F	Others
05H025	KMT2A rearranged	-	AML-M5A	23	F	Others
05H066	KMT2A rearranged	-	AML-M4	26	F	Others
06H088	KMT2A rearranged	-	AML-M1	28	M	Others
07H003	KMT2A rearranged	-	AML-M4	25	F	Others
07H045	KMT2A rearranged	-	AML-M5A	31	F	Others
08H139	KMT2A rearranged	-	AML-M5A	70	M	Others
09H018	KMT2A rearranged	-	AML-M0	56	M	Others
10H031	KMT2A rearranged	-	AML-M5B	25	F	Others
09H010	KMT2A rearranged	-	AML-M5A	20	M	Others
09H032	KMT2A rearranged	-	AML-M5A	54	M	Others
10H127	KMT2A rearranged	-	AML-M5A	36	M	Others
11H232	Deletion 7q (not complex)	-	AML-M1	58	F	Others
14H059	Deletion 7q (not complex)	-	AML-M1	56	M	Others
04H096	Monosomy 7	-	AML-M0	72	M	Others
05H193	Monosomy 7	-	Not classifiable by FAB criteria	62	M	Others
15H045	Monosomy 7	-	AML-M2	60	F	Others
18H146	Monosomy 7	-	AML-M1	65	M	Others
18H152	Monosomy 7	-	AML-M2	61	M	Others
06H038	t(6;9)	-	AML-M4	38	M	Others
13H101	t(6;9)	-	AML-M2	24	M	Others
03H083	t(8;21)	-	AML-M2	61	M	Others
05H042	t(8;21)	-	AML-M2	37	M	Others
07H137	t(8;21)	-	AML-M1	71	F	Others
09H040	t(8;21)	-	AML-M1	35	M	Others
10H119	t(8;21)	-	AML-M2	36	M	Others
12H045	t(8;21)	-	AML-M2	58	M	Others
07H155	Trisomy/tetrasomy 8	-	AML-M5B	71	M	Others
08H063	Trisomy/tetrasomy 8	-	AML-M5A	76	M	Others
08H108	Trisomy/tetrasomy 8	-	AML-M5A	72	M	Others
15H063	Trisomy/tetrasomy 8	-	Not classifiable by FAB criteria	28	F	Others
17H059	Trisomy/tetrasomy 8	-	AML-M5A	52	F	Others
07H063	Intermediate abnormal karyotype (except isolated trisomy/tetrasomy 8)	NPM1 mutated	AML-M5A	79	F	Others
09H024	Intermediate abnormal karyotype (except isolated trisomy/tetrasomy 8)	NPM1 mutated	AML-M5A	47	M	Others

11H217	Intermediate abnormal karyotype (except isolated trisomy/tetrasomy 8)	NPM1 mutated	Not classifiable by FAB criteria	63	M	Others
12H079	Intermediate abnormal karyotype (except isolated trisomy/tetrasomy 8)	NPM1 mutated	AML-M1	44	M	Others
07H107	Normal karyotype	NPM1 mutated	AML-M1	62	F	Others
09H070	Normal karyotype	NPM1 mutated	AML-M1	72	M	Others
12H033	Normal karyotype	NPM1 mutated	Not classifiable by FAB criteria	65	M	Others
12H091	Normal karyotype	NPM1 mutated	AML-M1	62	F	Others
13H053	Normal karyotype	NPM1 mutated	AML-M4	60	F	Others
14H020	Normal karyotype	NPM1 mutated	AML-M1	49	F	Others
07H042	Normal karyotype	NPM1, DNMT3A and FLT3(ITD) mutated	Not classifiable by FAB criteria	23	M	NK triple mutated
07H062	Normal karyotype	NPM1, DNMT3A and FLT3(ITD) mutated	AML-M1	58	M	NK triple mutated
07H134	Normal karyotype	NPM1, DNMT3A and FLT3(ITD) mutated	AML-M5	64	M	NK triple mutated
08H053	Normal karyotype	NPM1, DNMT3A and FLT3(ITD) mutated	AML-M1	76	F	NK triple mutated
08H089	Normal karyotype	NPM1, DNMT3A and FLT3(ITD) mutated	AML-M5	68	M	NK triple mutated
09H043	Normal karyotype	NPM1, DNMT3A and FLT3(ITD) mutated	AML-M1	53	M	NK triple mutated
10H092	Normal karyotype	NPM1, DNMT3A and FLT3(ITD) mutated	AML-M1	69	F	NK triple mutated
10H095	Normal karyotype	NPM1, DNMT3A and FLT3(ITD) mutated	AML-M1	65	F	NK triple mutated
10H166	Normal karyotype	NPM1, DNMT3A and FLT3(ITD) mutated	Not classifiable by FAB criteria	63	M	NK triple mutated
12H010	Normal karyotype	NPM1, DNMT3A and FLT3(ITD) mutated	AML-M1	65	M	NK triple mutated
12H056	Normal karyotype	NPM1, DNMT3A and FLT3(ITD) mutated	AML-M1	62	M	NK triple mutated
14H007	Normal karyotype	NPM1, DNMT3A and FLT3(ITD) mutated	AML-M2	53	F	NK triple mutated
04H001	Intermediate abnormal karyotype (except isolated trisomy/tetrasomy 8)	RUNX1 mutated	AML-M1	63	M	Others
04H055	Intermediate abnormal karyotype (except isolated trisomy/tetrasomy 8)	RUNX1 mutated	AML-M1	72	M	Others
08H065	Intermediate abnormal karyotype (except isolated trisomy/tetrasomy 8)	RUNX1 mutated	AML-M1	27	M	Others
13H139	Intermediate abnormal karyotype (except isolated trisomy/tetrasomy 8)	RUNX1 mutated	AML-M1	58	M	Others
14H027	Intermediate abnormal karyotype (except isolated trisomy/tetrasomy 8)	RUNX1 mutated	AML-M0	78	M	Others
06H133	Normal karyotype	RUNX1 mutated	AML-M0	45	F	Others
07H152	Normal karyotype	RUNX1 mutated	AML-M1	45	M	Others
08H087	Normal karyotype	RUNX1 mutated	AML-M2	46	F	Others
12H021	Normal karyotype	RUNX1 mutated	AML-M1	59	M	Others
12H175	Normal karyotype	RUNX1 mutated	AML-M1	59	F	Others

Supplement Table 1: Samples used for Surfaceome cohort.

Gene_name	p.adj	log2FC	Uniprot ID	Therapeutic Antibody (FDA approved or in clinical trial)	Nkt-AML median intensity
ADGRG1	0.0268	13.6	Q9Y653	N	22.67732
EPHB3	0.167	9.26	P54753	N	20.71788
DSC2	0.376	7.98	Q02487	N	24.27284
CNNM4	0.326	7.81	Q6P4Q7	N	24.19016
SLC26A6	0.35	7.2	Q9BXS9	N	22.86644
MYOF	0.451	7.16	Q9NZM1	N	24.39422
SLC7A6	0.469	6.6	Q92536	N	23.56386
P2RX4	0.484	6.51	Q99571	N	19.9513
KIAA0319L	0.533	5.99	Q8IZA0	N	21.36032
ACVR1	0.578	5.35	Q04771	Prafnosbart	22.37915
SIGLEC7	0.586	5.23	Q9Y286	N	24.28393
CD300LF	0.53	5.05	Q8TDQ1	N	21.88094
ELANE	0.627	4.95	P08246	N	23.36587
GP1BB	0.61	4.8	P13224	N	23.86008
BTN3A3	0.632	4.69	O00478	N	22.51284
SLC33A1	0.689	4.65	O00400	N	22.64467
ATP7A	0.716	4.64	Q04656	N	22.37025
TMX2	0.659	4.61	Q9Y320	N	21.8069
P2RX1	0.689	4.19	P51575	N	22.29732
SUSD1	0.739	3.92	Q6UWL2	N	21.58456
PLXDC2	0.608	3.92	Q6UX71	N	24.61949
IGHG1	0.775	3.8	P01857	N	21.2471
FLT3	0.627	3.8	P36888	Emirodatamab	23.32715
CD81	0.775	3.8	P60033	N	19.35783
SLC2A1	0.653	3.77	P11166	N	22.46957
PLSCR1	0.786	3.55	O15162	N	21.77424
DAGLB	0.739	3.52	Q8NCG7	N	23.16984
CTSG	0.781	3.5	P08311	N	25.69622
SMPDL3B	0.781	3.5	Q92485	N	23.32212
CD69	0.805	3.41	Q07108	N	23.45701
ATP1B1	0.781	3.28	P05026	N	22.75251
ADGRG5	0.662	3.16	Q8IZF4	N	26.07979
RELL1	0.81	3.02	Q8IUW5	N	23.49144
HAVCR2	0.857	2.99	Q8TDQ0	Cobolimab	22.11657
SLC29A1	0.729	2.91	Q99808	N	24.52425
CPD	0.787	2.78	O75976	N	22.32611
GGT5	0.786	2.71	P36269	N	26.09083
C1QBP	0.781	2.62	Q07021	N	22.01802
SLC16A1	0.735	2.58	P53985	N	25.46497
LRRC8D	0.781	2.57	Q7L1W4	N	23.25609
PTGFRN	0.877	2.55	Q9P2B2	N	21.0859
SORT1	0.848	2.54	Q99523	Latozinemab	22.97056
DYSF	0.858	2.45	O75923	N	22.91132
SCARF1	0.858	2.39	Q14162	N	24.36239
SLC19A1	0.858	2.29	P41440	N	23.26727
CYBB	0.782	2.25	P04839	N	22.67521
IL3RA	0.729	2.25	P26951	Flotetuzumab	26.15439
CD47	0.729	2.21	Q08722	Lemzoparlimab	27.77183
GP1BA	0.879	2.16	P07359	N	22.43346
SIGLEC9	0.877	2.1	Q9Y336	N	21.64285
ATP2B1	0.877	2.07	P20020	N	23.09565
ADGRE2	0.775	2.07	Q9UHX3	N	25.80314
ALG10	0.861	2.06	Q5BKT4	N	23.75172
TMEM167A	0.877	2.03	Q8TBQ9	N	19.9513
ALCAM	0.738	1.96	Q13740	N	24.18141

CD302	0.893	1.95	Q8IX05	N	23.04217
JAM3	0.893	1.92	Q9BX67	N	22.27406
PLAUR	0.877	1.87	Q03405	N	24.62776
CSF2RA	0.877	1.86	P15509	Mavrilimumab	23.33339
MRC1	0.881	1.83	P22897	N	24.10379
CD82	0.877	1.82	P27701	N	25.71068
IL1RAP	0.656	1.8	Q9NPH3	Nadunolimab	27.14753
EVI2B	0.893	1.77	P34910	N	25.60118
VSIR	0.0968	1.75	Q9H7M9	Onvatilimab	29.04284
CD36	0.781	1.74	P16671	N	26.02361
SCARB1	0.911	1.74	Q8WTV0	N	22.60214
LRRC8C	0.853	1.69	Q8TDW0	N	24.51845
SLC1A4	0.872	1.67	P43007	N	25.04456
SLC39A6	0.879	1.66	Q13433	Ladiratuzumab	23.97066
MRC2	0.893	1.66	Q9UBG0	N	23.74657
LAMP1	0.858	1.65	P11279	N	23.97339
ORAI1	0.786	1.65	Q96D31	N	25.36225
FGG	0.929	1.57	P02679	N	20.22066
VNN1	0.929	1.48	O95497	N	23.66169
TM9SF4	0.928	1.38	Q92544	N	24.65792
TMEM173	0.672	1.35	Q86WV6	N	25.63818
ANO10	0.929	1.33	Q9NW15	N	23.50544
SLC16A7	0.929	1.28	O60669	N	22.36559
NOTCH1	0.929	1.24	P46531	Brontictuzumab	23.62556
P2RY8	0.929	1.19	Q86VZ1	N	23.53745
FLOT1	0.929	1.15	O75955	N	21.53574
ITPR2	0.929	1.14	Q14571	N	22.6976
ABCC1	0.778	1.11	P33527	N	26.75941
TRPV2	0.856	1.11	Q9Y5S1	N	26.39603
LNPEP	0.343	1.1	Q9UIQ6	N	25.80656
LMAN2	0.781	1.08	Q12907	N	24.50891
COMT	0.877	1.06	P21964	N	27.53257
GOT2	0.311	1.03	P00505	N	26.98429

Supplement Table 2: 88 surface proteins enriched in NKt-AML (n=12) vs others (n=88)

group	IL1RAP	ADGRG5	CTSG
CD34+CD45RA-	7.13	11.85	3.09
CD34+CD45RA-	6.85	4.22	1.28
CD34+	21.07	4.33	40.13
CD34+	19.13	4.52	50.59
CD34+	14.23	5.64	26.47
CD34+CD45RA-	9.26	2.62	3.28
CD34+	16.16	8.25	61.08
CD34+CD45RA-	5.5	5.32	14.95
CD34+CD45RA-	21.76	2.85	0.86
CD34+CD45RA-	14.3	3.28	2.27
CD34+CD45RA-	7.8	7.66	6.71
CD34+CD45RA-	11.6	4.42	1.52
CD34+CD45RA-	15.77	3.42	3.89
CD34+CD45RA-	9.51	2.35	1.82
CD34+CD45RA-	18.83	3.05	1.55
CD34+CD45RA-	9.67	2.56	2.41
WBC	39.28	10.98	2.15
WBC	69.95	9.29	1.52
WBC	37.5	16.07	1.62
B-cells	2.37	15.78	0
B-cells	1.35	15.89	0
B-cells	1.44	15.95	0.05
B-cells	3.04	22.21	0
B-cells	1.81	15.4	0.08
Granulocytes	118.65	1.12	3.69
Granulocytes	111.21	2.71	0.92
Granulocytes	83.59	1.95	2.21
Granulocytes	127.32	1.14	8.4
Granulocytes	89.73	1.51	3.98
Monocytes	9.34	0.28	0.06
Monocytes	9.74	0.37	0.13
Monocytes	10.78	0.4	4.62
Monocytes	10.51	0.72	0.11
Monocytes	7.77	0.56	0
peripheral blood CD34+ cells	4.95	7.35	4.46
T-cells	3.19	9.1	0.05
T-cells	2.9	9.71	0
T-cells	2.97	6.98	0.12
T-cells	4.4	11.73	0
T-cells	3.3	20.6	0.12
Gran-V	65.56	0.1	212.59
Gran-IV	4.55	0.99	245.32
Gran-I	10.04	8.1	817.54
Gran-III	4.42	0.1	2903.22
Gran-II	6.64	0.29	7035.12
Ery-I	2.6	1.04	545.3
Ery-II	1	2.51	225.97
Ery-IV	18.41	2.78	88.12
Gran-I	19.69	3.95	2968.42
Pre-B-I	1.13	0.53	9.99
Pre-B-II	1.83	0.72	4.78
Gran-V	45.1	0.03	5.49
Ery-I	3.59	0.73	248.2
Ery-II	0.46	0.01	16.66
Ery-III	0.5	0.03	27.94
Ery-IV	46.85	0.95	887.37

Gran-IV	4.3	0.16	6.17
Gran-III	8.08	0.03	53.8
Gran-I	23.24	1.26	1385.24
Pre-B-I	2.98	0.18	15.95
Pre-B-II	3.09	0.12	5.46
Gran-II	16.86	0.1	5512.23
Gran-I	10.36	1.22	566.52

Supplement Table 3 : Normal expression for the three target

sample	NPM1 mutation	FLT3-ITD mutation	DNMT3A mutation	cytogenetic_group	cytogenetic_risk
01H001	0	0	0	KMT2A rearranged	adverse
01H002	0	1	0	t(15;17)(q24;q21)/PML-RARA (Irrespective of additional cytogenetic abnormalities)	favorable
02H003	0	0	0	Intermediate abnormal karyotype (except isolated trisomy/tetrasomy 8)	intermediate
02H009	1	1	0	Normal karyotype	intermediate
02H017	NA	0	NA	KMT2A rearranged	adverse
02H025	1	1	1	Intermediate abnormal karyotype (except isolated trisomy/tetrasomy 8)	intermediate
02H026	1	0	0	Complex (3 and more chromosomal abnormalities)	adverse
02H032	0	0	0	KMT2A rearranged	adverse
02H033	0	0	1	KMT2A rearranged	adverse
02H043	1	1	0	Intermediate abnormal karyotype (except isolated trisomy/tetrasomy 8)	intermediate
02H046	0	1	0	t(15;17)(q24;q21)/PML-RARA (Irrespective of additional cytogenetic abnormalities)	favorable
02H053	1	1	0	Normal karyotype	intermediate
02H060	0	0	0	Intermediate abnormal karyotype (except isolated trisomy/tetrasomy 8)	intermediate
02H066	1	1	1	Normal karyotype	intermediate
02H080	0	0	0	Normal karyotype	intermediate
03H004	1	1	1	Normal karyotype	intermediate
03H016	1	1	1	Normal karyotype	intermediate
03H022	0	0	0	Complex (3 and more chromosomal abnormalities)	adverse
03H024	0	0	0	Trisomy/tetrasomy 8 (isolated)	intermediate
03H028	0	0	0	Complex (3 and more chromosomal abnormalities)	adverse
03H030	0	0	0	Complex (3 and more chromosomal abnormalities)	adverse
03H033	1	1	0	Normal karyotype	intermediate
03H036	0	0	0	Monosomy 5/ 5q-/Monosomy 7/ 7q- (less than 3 chromosomal abnormalities)	intermediate
03H041	0	1	0	NUP98-NSD1(normal karyotype)	intermediate
03H049	1	1	0	Normal karyotype	intermediate
03H052	1	1	1	Normal karyotype	intermediate
03H060	0	0	0	Intermediate abnormal karyotype (except isolated trisomy/tetrasomy 8)	intermediate
03H065	0	0	0	t(8;21)(q22;q22)/RUNX1-RUNX1T1 (Irrespective of additional cytogenetic abnormalities)	favorable
03H067	0	0	0	KMT2A rearranged	adverse
03H070	0	1	0	t(15;17)(q24;q21)/PML-RARA (Irrespective of additional cytogenetic abnormalities)	favorable
03H081	0	0	1	Trisomy/tetrasomy 8 (isolated)	intermediate
03H083	0	0	1	t(8;21)(q22;q22)/RUNX1-RUNX1T1 (Irrespective of additional cytogenetic abnormalities)	favorable
03H090	1	1	1	Complex (3 and more chromosomal abnormalities)	adverse
03H092	0	1	0	Normal karyotype	intermediate
03H094	0	0	0	Complex (3 and more chromosomal abnormalities)	adverse
03H095	0	0	0	inv(16)(p13.1q22)/t(16;16)(p13.1;q22)/CBFB-MYH11 (Irrespective of additional cytogenetic abnormalities)	favorable
03H097	0	0	0	Normal karyotype	intermediate
03H109	0	0	0	inv(16)(p13.1q22)/t(16;16)(p13.1;q22)/CBFB-MYH11 (Irrespective of additional cytogenetic abnormalities)	favorable
03H112	NA	1	NA	inv(16)(p13.1q22)/t(16;16)(p13.1;q22)/CBFB-MYH11 (Irrespective of additional cytogenetic abnormalities)	favorable
03H116	1	1	0	Normal karyotype	intermediate
03H119	0	0	0	Normal karyotype	intermediate
04H001	0	0	0	Intermediate abnormal karyotype (except isolated trisomy/tetrasomy 8)	intermediate
04H006	1	1	1	Normal karyotype	intermediate
04H011	0	0	0	Normal karyotype	intermediate
04H017	0	0	0	Complex (3 and more chromosomal abnormalities)	adverse
04H021	0	0	1	Normal karyotype	intermediate
04H024	1	0	1	Normal karyotype	intermediate
04H025	1	1	0	Normal karyotype	intermediate
04H030	0	1	0	inv(16)(p13.1q22)/t(16;16)(p13.1;q22)/CBFB-MYH11 (Irrespective of additional cytogenetic abnormalities)	favorable
04H039	NA	1	NA	Normal karyotype	intermediate
04H041	0	0	0	KMT2A rearranged	adverse
04H048	0	0	0	Intermediate abnormal karyotype (except isolated trisomy/tetrasomy 8)	intermediate
04H050	0	0	0	MECOM rearranged	adverse
04H054	0	1	0	t(6;9)(p23;q34) (Irrespective of additional cytogenetic abnormalities)	adverse
04H055	0	1	0	Intermediate abnormal karyotype (except isolated trisomy/tetrasomy 8)	intermediate
04H061	0	0	0	inv(16)(p13.1q22)/t(16;16)(p13.1;q22)/CBFB-MYH11 (Irrespective of additional cytogenetic abnormalities)	favorable
04H063	0	0	0	MECOM rearranged	adverse
04H068	1	0	1	Normal karyotype	intermediate
04H078	1	0	0	Normal karyotype	intermediate
04H080	0	0	0	KMT2A rearranged	intermediate
04H084	1	0	1	Normal karyotype	intermediate
04H091	0	0	0	inv(16)(p13.1q22)/t(16;16)(p13.1;q22)/CBFB-MYH11 (Irrespective of additional cytogenetic abnormalities)	favorable
04H094	1	1	1	Normal karyotype	intermediate
04H096	0	0	0	Monosomy 5/ 5q-/Monosomy 7/ 7q- (less than 3 chromosomal abnormalities)	adverse
04H100	0	0	0	Normal karyotype	intermediate
04H101	0	0	0	Complex (3 and more chromosomal abnormalities)	adverse
04H103	0	0	0	Intermediate abnormal karyotype (except isolated trisomy/tetrasomy 8)	intermediate
04H107	0	0	0	Normal karyotype	intermediate
04H108	1	1	0	Normal karyotype	intermediate
04H111	0	0	0	Intermediate abnormal karyotype (except isolated trisomy/tetrasomy 8)	intermediate
04H112	1	1	1	Normal karyotype	intermediate
04H115	0	0	0	Complex (3 and more chromosomal abnormalities)	adverse
04H117	1	0	0	Normal karyotype	intermediate
04H118	1	0	0	Intermediate abnormal karyotype (except isolated trisomy/tetrasomy 8)	intermediate
04H120	0	0	0	Monosomy 5/ 5q-/Monosomy 7/ 7q- (less than 3 chromosomal abnormalities)	adverse
04H121	0	0	0	KMT2A rearranged	adverse
04H123	0	0	1	t(15;17)(q24;q21)/PML-RARA (Irrespective of additional cytogenetic abnormalities)	favorable
04H127	0	0	0	Complex (3 and more chromosomal abnormalities)	adverse
04H132	0	0	0	Complex (3 and more chromosomal abnormalities)	adverse
04H133	1	1	1	Normal karyotype	intermediate

04H135	0	0	1	Intermediate abnormal karyotype (except isolated trisomy/tetrasomy 8)	intermediate
04H138	0	0	0	Trisomy/tetrasomy 8 (isolated)	intermediate
04H140	1	1	0	Normal karyotype	intermediate
04H141	0	0	1	Normal karyotype	intermediate
05H004	0	0	1	Monosomy 5/ 5q-/Monosomy 7/ 7q- (less than 3 chromosomal abnormalities)	adverse
05H008	1	0	1	Normal karyotype	intermediate
05H013	0	0	0	Intermediate abnormal karyotype (except isolated trisomy/tetrasomy 8)	intermediate
05H022	0	0	0	Normal karyotype	intermediate
05H025	0	0	0	KMT2A rearranged	adverse
05H030	1	1	1	Normal karyotype	intermediate
05H031	0	0	0	Monosomy 5/ 5q-/Monosomy 7/ 7q- (less than 3 chromosomal abnormalities)	adverse
05H033	1	1	0	Normal karyotype	intermediate
05H034	0	0	0	Monosomy 5/ 5q-/Monosomy 7/ 7q- (less than 3 chromosomal abnormalities)	adverse
05H039	1	0	1	Normal karyotype	intermediate
05H042	0	0	0	t(8;21)(q22;q22)/RUNX1-RUNX1T1 (Irrespective of additional cytogenetic abnormalities)	favorable
05H046	0	0	0	Intermediate abnormal karyotype (except isolated trisomy/tetrasomy 8)	intermediate
05H050	0	1	1	Normal karyotype	intermediate
05H054	0	0	1	Trisomy/tetrasomy 8 (isolated)	intermediate
05H056	1	0	0	Normal karyotype	intermediate
05H065	1	0	1	Normal karyotype	intermediate
05H066	NA	1	NA	KMT2A rearranged	adverse
05H072	0	1	0	t(15;17)(q24;q21)/PML-RARA (Irrespective of additional cytogenetic abnormalities)	favorable
05H073	1	1	0	Normal karyotype	intermediate
05H078	0	0	1	Normal karyotype	intermediate
05H090	0	0	0	inv(16)(p13.1q22)/t(16;16)(p13.1;q22)/CBFB-MYH11 (Irrespective of additional cytogenetic abnormalities)	favorable
05H094	1	0	0	Normal karyotype	intermediate
05H095	0	0	0	Normal karyotype	intermediate
05H097	1	0	0	Normal karyotype	intermediate
05H099	0	0	0	inv(16)(p13.1q22)/t(16;16)(p13.1;q22)/CBFB-MYH11 (Irrespective of additional cytogenetic abnormalities)	favorable
05H108	1	0	0	Normal karyotype	intermediate
05H111	0	1	0	Normal karyotype	intermediate
05H113	0	0	0	inv(16)(p13.1q22)/t(16;16)(p13.1;q22)/CBFB-MYH11 (Irrespective of additional cytogenetic abnormalities)	favorable
05H118	0	0	0	t(8;21)(q22;q22)/RUNX1-RUNX1T1 (Irrespective of additional cytogenetic abnormalities)	favorable
05H127	0	0	0	inv(16)(p13.1q22)/t(16;16)(p13.1;q22)/CBFB-MYH11 (Irrespective of additional cytogenetic abnormalities)	favorable
05H128	0	0	0	KMT2A rearranged	adverse
05H136	0	0	0	inv(16)(p13.1q22)/t(16;16)(p13.1;q22)/CBFB-MYH11 (Irrespective of additional cytogenetic abnormalities)	favorable
05H141	0	0	0	Complex (3 and more chromosomal abnormalities)	adverse
05H143	1	1	1	Normal karyotype	intermediate
05H149	0	0	1	Normal karyotype	intermediate
05H155	1	1	0	Normal karyotype	intermediate
05H160	0	0	0	Normal karyotype	intermediate
05H163	0	0	0	NUP98-NSD1(normal karyotype)	intermediate
05H176	0	0	0	Complex (3 and more chromosomal abnormalities)	adverse
05H179	0	0	0	MECOM rearranged	adverse
05H180	0	0	0	Complex (3 and more chromosomal abnormalities)	adverse
05H181	1	1	0	Normal karyotype	intermediate
05H184	0	0	0	t(8;21)(q22;q22)/RUNX1-RUNX1T1 (Irrespective of additional cytogenetic abnormalities)	favorable
05H186	1	0	1	Normal karyotype	intermediate
05H192	0	0	0	Normal karyotype	intermediate
05H193	0	0	0	Monosomy 5/ 5q-/Monosomy 7/ 7q- (less than 3 chromosomal abnormalities)	adverse
05H195	1	1	0	Normal karyotype	intermediate
06H004	0	0	0	Complex (3 and more chromosomal abnormalities)	adverse
06H010	1	1	1	Normal karyotype	intermediate
06H011	1	0	1	Normal karyotype	intermediate
06H014	0	0	1	Monosomy 5/ 5q-/Monosomy 7/ 7q- (less than 3 chromosomal abnormalities)	adverse
06H016	0	0	1	Complex (3 and more chromosomal abnormalities)	adverse
06H019	0	0	0	Trisomy/tetrasomy 8 (isolated)	intermediate
06H020	0	0	0	inv(16)(p13.1q22)/t(16;16)(p13.1;q22)/CBFB-MYH11 (Irrespective of additional cytogenetic abnormalities)	favorable
06H021	1	1	1	Normal karyotype	intermediate
06H026	0	1	0	Intermediate abnormal karyotype (except isolated trisomy/tetrasomy 8)	intermediate
06H028	1	0	0	Normal karyotype	intermediate
06H029	0	0	0	Complex (3 and more chromosomal abnormalities)	adverse
06H030	1	0	1	Normal karyotype	intermediate
06H035	0	0	0	t(8;21)(q22;q22)/RUNX1-RUNX1T1 (Irrespective of additional cytogenetic abnormalities)	favorable
06H038	NA	1	NA	t(6;9)(p23;q34) (Irrespective of additional cytogenetic abnormalities)	adverse
06H045	0	0	0	Intermediate abnormal karyotype (except isolated trisomy/tetrasomy 8)	intermediate
06H048	0	0	1	Trisomy/tetrasomy 8 (isolated)	intermediate
06H051	0	0	0	Normal karyotype	intermediate
06H054	0	0	0	Normal karyotype	intermediate
06H061	1	0	1	Normal karyotype	intermediate
06H063	0	0	0	inv(16)(p13.1q22)/t(16;16)(p13.1;q22)/CBFB-MYH11 (Irrespective of additional cytogenetic abnormalities)	favorable
06H066	NA	0	NA	KMT2A rearranged	adverse
06H073	0	0	1	KMT2A rearranged	adverse
06H074	1	0	0	Complex (3 and more chromosomal abnormalities)	adverse
06H075	0	0	0	Complex (3 and more chromosomal abnormalities)	adverse
06H077	0	0	0	KMT2A rearranged	adverse
06H088	0	0	0	KMT2A rearranged	adverse
06H089	0	0	0	Intermediate abnormal karyotype (except isolated trisomy/tetrasomy 8)	intermediate
06H090	0	1	1	Normal karyotype	intermediate
06H099	1	1	0	Normal karyotype	intermediate

06H103	1	0	0	Normal karyotype	intermediate
06H106	1	0	0	Intermediate abnormal karyotype (except isolated trisomy/tetrasomy 8)	intermediate
06H112	1	0	0	Normal karyotype	intermediate
06H115	0	0	0	inv(16)(p13.1q22)/t(16;16)(p13.1;q22)/CBFB-MYH11 (Irrespective of additional cytogenetic abnormalities)	favorable
06H117	0	0	0	KMT2A rearranged	adverse
06H122	0	0	0	Normal karyotype	intermediate
06H133	0	1	0	Normal karyotype	intermediate
06H135	0	0	0	Complex (3 and more chromosomal abnormalities)	adverse
06H143	0	1	0	Intermediate abnormal karyotype (except isolated trisomy/tetrasomy 8)	intermediate
06H144	1	0	1	Normal karyotype	intermediate
06H146	0	1	0	Normal karyotype	intermediate
06H151	0	0	0	inv(16)(p13.1q22)/t(16;16)(p13.1;q22)/CBFB-MYH11 (Irrespective of additional cytogenetic abnormalities)	favorable
06H152	0	0	0	KMT2A rearranged	adverse
07H003	0	1	0	KMT2A rearranged	adverse
07H005	1	1	0	Normal karyotype	intermediate
07H006	1	0	1	Normal karyotype	intermediate
07H009	1	1	1	Normal karyotype	intermediate
07H019	0	0	0	Trisomy/tetrasomy 8 (isolated)	intermediate
07H020	0	0	0	Normal karyotype	intermediate
07H024	0	0	0	Complex (3 and more chromosomal abnormalities)	adverse
07H034	0	0	0	Complex (3 and more chromosomal abnormalities)	adverse
07H038	0	0	0	Complex (3 and more chromosomal abnormalities)	adverse
07H041	0	0	0	KMT2A rearranged	intermediate
07H042	1	1	1	Normal karyotype	intermediate
07H043	NA	0	NA	t(15;17)(q24;q21)/PML-RARA (Irrespective of additional cytogenetic abnormalities)	favorable
07H045	0	0	0	KMT2A rearranged	adverse
07H052	NA	0	NA	t(15;17)(q24;q21)/PML-RARA (Irrespective of additional cytogenetic abnormalities)	favorable
07H055	1	0	1	Normal karyotype	intermediate
07H059	0	0	0	Normal karyotype	intermediate
07H060	1	0	1	Intermediate abnormal karyotype (except isolated trisomy/tetrasomy 8)	intermediate
07H061	0	0	0	Complex (3 and more chromosomal abnormalities)	adverse
07H062	1	1	1	Normal karyotype	intermediate
07H063	1	0	0	Intermediate abnormal karyotype (except isolated trisomy/tetrasomy 8)	intermediate
07H069	0	0	0	Intermediate abnormal karyotype (except isolated trisomy/tetrasomy 8)	intermediate
07H073	0	0	0	KMT2A rearranged	adverse
07H082	0	0	1	t(15;17)(q24;q21)/PML-RARA (Irrespective of additional cytogenetic abnormalities)	favorable
07H083	0	1	0	Normal karyotype	intermediate
07H089	1	1	0	Normal karyotype	intermediate
07H091	1	1	1	Normal karyotype	intermediate
07H095	1	1	1	Normal karyotype	intermediate
07H098	0	1	0	Intermediate abnormal karyotype (except isolated trisomy/tetrasomy 8)	intermediate
07H099	0	0	0	inv(16)(p13.1q22)/t(16;16)(p13.1;q22)/CBFB-MYH11 (Irrespective of additional cytogenetic abnormalities)	favorable
07H106	1	0	1	Normal karyotype	intermediate
07H107	1	0	0	Normal karyotype	intermediate
07H112	1	0	0	Normal karyotype	intermediate
07H117	1	0	0	Normal karyotype	intermediate
07H124	0	0	0	Intermediate abnormal karyotype (except isolated trisomy/tetrasomy 8)	intermediate
07H125	1	1	0	Normal karyotype	intermediate
07H131	0	0	0	Normal karyotype	intermediate
07H133	1	1	0	Intermediate abnormal karyotype (except isolated trisomy/tetrasomy 8)	intermediate
07H134	1	1	1	Normal karyotype	intermediate
07H135	1	1	1	Normal karyotype	intermediate
07H137	0	0	0	t(8;21)(q22;q22)/RUNX1-RUNX1T1 (Irrespective of additional cytogenetic abnormalities)	favorable
07H138	1	0	1	Normal karyotype	intermediate
07H142	0	0	0	Complex (3 and more chromosomal abnormalities)	adverse
07H144	0	0	0	inv(16)(p13.1q22)/t(16;16)(p13.1;q22)/CBFB-MYH11 (Irrespective of additional cytogenetic abnormalities)	favorable
07H148	0	0	0	Complex (3 and more chromosomal abnormalities)	adverse
07H151	1	1	0	Intermediate abnormal karyotype (except isolated trisomy/tetrasomy 8)	intermediate
07H152	0	1	0	Normal karyotype	intermediate
07H155	0	0	0	Trisomy/tetrasomy 8 (isolated)	intermediate
07H156	0	1	0	Complex (3 and more chromosomal abnormalities)	adverse
07H158	1	1	0	Normal karyotype	intermediate
07H160	0	0	0	KMT2A rearranged	adverse
08H004	1	0	1	Normal karyotype	intermediate
08H011	1	0	0	Trisomy/tetrasomy 8 (isolated)	intermediate
08H012	0	1	1	Intermediate abnormal karyotype (except isolated trisomy/tetrasomy 8)	intermediate
08H018	0	0	0	Complex (3 and more chromosomal abnormalities)	adverse
08H021	0	0	0	KMT2A rearranged	intermediate
08H022	0	0	0	Complex (3 and more chromosomal abnormalities)	adverse
08H033	NA	0	NA	Complex (3 and more chromosomal abnormalities)	adverse
08H034	0	0	0	t(8;21)(q22;q22)/RUNX1-RUNX1T1 (Irrespective of additional cytogenetic abnormalities)	favorable
08H042	0	0	0	t(8;21)(q22;q22)/RUNX1-RUNX1T1 (Irrespective of additional cytogenetic abnormalities)	favorable
08H046	1	0	0	Normal karyotype	intermediate
08H048	0	1	0	Normal karyotype	intermediate
08H049	0	1	0	NUP98-NSD1(normal karyotype)	intermediate
08H050	1	0	1	Normal karyotype	intermediate
08H053	1	1	1	Normal karyotype	intermediate
08H054	0	0	0	Complex (3 and more chromosomal abnormalities)	adverse
08H055	1	0	0	Trisomy/tetrasomy 8 (isolated)	intermediate
08H056	1	1	0	Intermediate abnormal karyotype (except isolated trisomy/tetrasomy 8)	intermediate

08H060	0	0	0	Complex (3 and more chromosomal abnormalities)	adverse
08H062	0	0	1	Intermediate abnormal karyotype (except isolated trisomy/tetrasomy 8)	intermediate
08H063	0	0	0	Trisomy/tetrasomy 8 (isolated)	intermediate
08H065	0	0	0	Intermediate abnormal karyotype (except isolated trisomy/tetrasomy 8)	intermediate
08H072	0	1	0	t(8;21)(q22;q22)/RUNX1-RUNX1T1 (Irrespective of additional cytogenetic abnormalities)	favorable
08H080	0	0	0	Normal karyotype	intermediate
08H081	0	0	0	inv(16)(p13.1q22)/t(16;16)(p13.1;q22)/CBFB-MYH11 (Irrespective of additional cytogenetic abnormalities)	favorable
08H082	1	1	1	Normal karyotype	intermediate
08H085	NA	0	NA	KMT2A rearranged	adverse
08H087	0	0	0	Normal karyotype	intermediate
08H088	0	0	0	Trisomy/tetrasomy 8 (isolated)	intermediate
08H089	1	1	1	Normal karyotype	intermediate
08H092	0	0	0	Germ cell tumor -associated hematologic malignancies_iso12p	adverse
08H099	0	0	0	inv(16)(p13.1q22)/t(16;16)(p13.1;q22)/CBFB-MYH11 (Irrespective of additional cytogenetic abnormalities)	favorable
08H104	1	0	0	Normal karyotype	intermediate
08H108	1	1	1	Trisomy/tetrasomy 8 (isolated)	intermediate
08H112	0	0	0	Normal karyotype	intermediate
08H113	1	1	1	Normal karyotype	intermediate
08H116	0	0	1	Monosomy 5/ 5q-/Monosomy 7/ 7q- (less than 3 chromosomal abnormalities)	intermediate
08H118	0	0	0	MECOM rearranged	adverse
08H129	0	0	0	KMT2A rearranged	adverse
08H137	0	0	1	Monosomy 5/ 5q-/Monosomy 7/ 7q- (less than 3 chromosomal abnormalities)	adverse
08H138	0	1	0	Normal karyotype	intermediate
08H139	0	0	0	KMT2A rearranged	adverse
09H002	1	1	1	Normal karyotype	intermediate
09H005	0	0	0	t(15;17)(q24;q21)/PML-RARA (Irrespective of additional cytogenetic abnormalities)	favorable
09H008	1	0	0	Normal karyotype	intermediate
09H010	NA	0	NA	KMT2A rearranged	intermediate
09H013	1	0	1	Normal karyotype	intermediate
09H015	0	0	0	Intermediate abnormal karyotype (except isolated trisomy/tetrasomy 8)	intermediate
09H016	NA	0	NA	inv(16)(p13.1q22)/t(16;16)(p13.1;q22)/CBFB-MYH11 (Irrespective of additional cytogenetic abnormalities)	favorable
09H018	0	0	0	KMT2A rearranged	adverse
09H022	0	0	0	Intermediate abnormal karyotype (except isolated trisomy/tetrasomy 8)	intermediate
09H024	1	0	0	Intermediate abnormal karyotype (except isolated trisomy/tetrasomy 8)	intermediate
09H026	1	1	1	Normal karyotype	intermediate
09H031	1	1	0	Normal karyotype	intermediate
09H032	0	0	0	KMT2A rearranged	intermediate
09H035	NA	0	NA	t(15;17)(q24;q21)/PML-RARA (Irrespective of additional cytogenetic abnormalities)	favorable
09H036	0	0	1	Normal karyotype	intermediate
09H040	0	0	0	t(8;21)(q22;q22)/RUNX1-RUNX1T1 (Irrespective of additional cytogenetic abnormalities)	favorable
09H042	1	1	1	Normal karyotype	intermediate
09H043	1	1	1	Normal karyotype	intermediate
09H045	0	0	0	Complex (3 and more chromosomal abnormalities)	adverse
09H046	0	0	1	Monosomy17/del17p (less than 3 chromosomal abnormalities)	adverse
09H048	1	1	1	Normal karyotype	intermediate
09H053	NA	0	NA	t(15;17)(q24;q21)/PML-RARA (Irrespective of additional cytogenetic abnormalities)	favorable
09H054	0	0	0	Complex (3 and more chromosomal abnormalities)	adverse
09H057	0	0	1	Complex (3 and more chromosomal abnormalities)	adverse
09H058	0	1	1	Normal karyotype	intermediate
09H060	0	0	0	Monosomy 5/ 5q-/Monosomy 7/ 7q- (less than 3 chromosomal abnormalities)	adverse
09H062	0	0	0	t(15;17)(q24;q21)/PML-RARA (Irrespective of additional cytogenetic abnormalities)	favorable
09H066	0	1	0	inv(16)(p13.1q22)/t(16;16)(p13.1;q22)/CBFB-MYH11 (Irrespective of additional cytogenetic abnormalities)	favorable
09H070	1	0	0	Normal karyotype	intermediate
09H073	0	0	0	t(15;17)(q24;q21)/PML-RARA (Irrespective of additional cytogenetic abnormalities)	favorable
09H078	0	0	0	Complex (3 and more chromosomal abnormalities)	adverse
09H079	0	0	0	Normal karyotype	intermediate
09H083	1	1	1	Normal karyotype	intermediate
09H084	0	0	0	Complex (3 and more chromosomal abnormalities)	adverse
09H085	0	0	0	Complex (3 and more chromosomal abnormalities)	adverse
09H088	1	0	1	Intermediate abnormal karyotype (except isolated trisomy/tetrasomy 8)	intermediate
09H089	0	0	0	Complex (3 and more chromosomal abnormalities)	adverse
09H090	1	0	0	Intermediate abnormal karyotype (except isolated trisomy/tetrasomy 8)	intermediate
09H092	0	0	0	Intermediate abnormal karyotype (except isolated trisomy/tetrasomy 8)	intermediate
09H094	1	1	1	Intermediate abnormal karyotype (except isolated trisomy/tetrasomy 8)	intermediate
09H098	0	0	0	KMT2A rearranged	intermediate
09H102	NA	0	NA	KMT2A rearranged	intermediate
09H106	0	1	1	Normal karyotype	intermediate
09H111	1	0	1	Normal karyotype	intermediate
09H113	0	0	1	Normal karyotype	intermediate
09H115	0	1	0	Normal karyotype	intermediate
09H117	0	0	0	Normal karyotype	intermediate
09H119	0	0	0	t(15;17)(q24;q21)/PML-RARA (Irrespective of additional cytogenetic abnormalities)	favorable
10H001	0	0	1	Intermediate abnormal karyotype (except isolated trisomy/tetrasomy 8)	intermediate
10H002	0	0	0	Normal karyotype	intermediate
10H005	0	0	0	Intermediate abnormal karyotype (except isolated trisomy/tetrasomy 8)	intermediate
10H007	0	1	0	Hyperdiploid numerical abnormalities only	Undetermined
10H008	0	0	0	inv(16)(p13.1q22)/t(16;16)(p13.1;q22)/CBFB-MYH11 (Irrespective of additional cytogenetic abnormalities)	favorable
10H014	0	0	1	Complex (3 and more chromosomal abnormalities)	adverse
10H015	0	0	0	Monosomy17/del17p (less than 3 chromosomal abnormalities)	adverse
10H017	NA	1	NA	t(15;17)(q24;q21)/PML-RARA (Irrespective of additional cytogenetic abnormalities)	favorable

10H022	0	0	0	KMT2A rearranged	intermediate
10H023	0	0	0	t(15;17)(q24;q21)/PML-RARA (Irrespective of additional cytogenetic abnormalities)	favorable
10H026	1	0	1	Normal karyotype	intermediate
10H029	1	0	0	Intermediate abnormal karyotype (except isolated trisomy/tetrasomy 8)	intermediate
10H030	0	0	0	t(8;21)(q22;q22)/RUNX1-RUNX1T1 (Irrespective of additional cytogenetic abnormalities)	favorable
10H031	0	0	0	KMT2A rearranged	adverse
10H034	NA	0	NA	Complex (3 and more chromosomal abnormalities)	adverse
10H038	0	0	0	NUP98-NSD1(normal karyotype)	intermediate
10H040	0	0	1	Intermediate abnormal karyotype (except isolated trisomy/tetrasomy 8)	intermediate
10H044	0	0	0	Complex (3 and more chromosomal abnormalities)	adverse
10H045	0	0	0	Normal karyotype	intermediate
10H046	0	0	0	MECOM rearranged	adverse
10H048	1	1	1	Undetermined	Undetermined
10H051	NA	0	NA	Complex (3 and more chromosomal abnormalities)	adverse
10H052	1	1	0	Normal karyotype	intermediate
10H053	0	1	0	t(15;17)(q24;q21)/PML-RARA (Irrespective of additional cytogenetic abnormalities)	favorable
10H055	0	0	0	KMT2A rearranged	intermediate
10H056	0	0	1	Normal karyotype	intermediate
10H058	0	0	0	KMT2A rearranged	intermediate
10H059	0	1	0	t(15;17)(q24;q21)/PML-RARA (Irrespective of additional cytogenetic abnormalities)	favorable
10H063	0	0	0	MECOM rearranged	adverse
10H066	0	0	0	Complex (3 and more chromosomal abnormalities)	adverse
10H068	1	1	1	Intermediate abnormal karyotype (except isolated trisomy/tetrasomy 8)	intermediate
10H070	0	1	0	Complex (3 and more chromosomal abnormalities)	adverse
10H072	1	0	1	Normal karyotype	intermediate
10H075	0	1	0	Normal karyotype	intermediate
10H078	0	1	1	Complex (3 and more chromosomal abnormalities)	adverse
10H087	0	0	0	Complex (3 and more chromosomal abnormalities)	adverse
10H089	0	0	0	Normal karyotype	intermediate
10H092	1	1	1	Normal karyotype	intermediate
10H095	1	1	1	Normal karyotype	intermediate
10H101	1	1	1	Normal karyotype	intermediate
10H106	0	1	0	t(15;17)(q24;q21)/PML-RARA (Irrespective of additional cytogenetic abnormalities)	favorable
10H107	0	0	0	MECOM rearranged	adverse
10H109	1	1	0	Intermediate abnormal karyotype (except isolated trisomy/tetrasomy 8)	intermediate
10H110	0	0	0	t(15;17)(q24;q21)/PML-RARA (Irrespective of additional cytogenetic abnormalities)	favorable
10H113	0	0	0	Complex (3 and more chromosomal abnormalities)	adverse
10H114	0	0	0	KMT2A rearranged	intermediate
10H115	1	1	0	Normal karyotype	intermediate
10H119	0	0	1	t(8;21)(q22;q22)/RUNX1-RUNX1T1 (Irrespective of additional cytogenetic abnormalities)	favorable
10H120	0	0	1	Normal karyotype	intermediate
10H125	0	0	0	Normal karyotype	intermediate
10H127	NA	0	NA	KMT2A rearranged	intermediate
10H130	0	0	0	Complex (3 and more chromosomal abnormalities)	adverse
10H136	0	0	0	Monosomy 5/ 5q-/Monosomy 7/ 7q- (less than 3 chromosomal abnormalities)	adverse
10H138	1	0	0	Normal karyotype	intermediate
10H149	0	1	0	Monosomy 5/ 5q-/Monosomy 7/ 7q- (less than 3 chromosomal abnormalities)	adverse
10H161	1	0	1	Trisomy/tetrasomy 8 (isolated)	intermediate
10H166	1	1	1	Normal karyotype	intermediate
10H173	0	0	0	Intermediate abnormal karyotype (except isolated trisomy/tetrasomy 8)	intermediate
10H174	0	0	0	Complex (3 and more chromosomal abnormalities)	adverse
11H001	0	0	0	Normal karyotype	intermediate
11H002	0	0	0	Complex (3 and more chromosomal abnormalities)	adverse
11H006	1	0	1	Normal karyotype	intermediate
11H008	0	1	0	Intermediate abnormal karyotype (except isolated trisomy/tetrasomy 8)	intermediate
11H009	1	1	1	Normal karyotype	intermediate
11H010	1	1	1	Normal karyotype	intermediate
11H014	1	0	0	Monosomy 5/ 5q-/Monosomy 7/ 7q- (less than 3 chromosomal abnormalities)	intermediate
11H015	0	0	0	Complex (3 and more chromosomal abnormalities)	adverse
11H017	0	0	0	Complex (3 and more chromosomal abnormalities)	adverse
11H019	1	1	0	Intermediate abnormal karyotype (except isolated trisomy/tetrasomy 8)	intermediate
11H021	0	1	0	Normal karyotype	intermediate
11H022	0	1	1	inv(16)(p13.1q22)/t(16;16)(p13.1;q22)/CBFB-MYH11 (Irrespective of additional cytogenetic abnormalities)	favorable
11H027	0	1	0	Intermediate abnormal karyotype (except isolated trisomy/tetrasomy 8)	intermediate
11H035	1	1	0	Intermediate abnormal karyotype (except isolated trisomy/tetrasomy 8)	intermediate
11H043	0	0	0	t(15;17)(q24;q21)/PML-RARA (Irrespective of additional cytogenetic abnormalities)	favorable
11H044	0	0	0	Complex (3 and more chromosomal abnormalities)	adverse
11H046	0	0	0	Normal karyotype	intermediate
11H058	1	1	0	Normal karyotype	intermediate
11H067	0	0	1	Normal karyotype	intermediate
11H072	1	1	1	Normal karyotype	intermediate
11H083	1	1	1	Normal karyotype	intermediate
11H089	0	0	1	Normal karyotype	intermediate
11H095	0	0	0	KMT2A rearranged	adverse
11H097	0	1	1	Monosomy 5/ 5q-/Monosomy 7/ 7q- (less than 3 chromosomal abnormalities)	adverse
11H103	0	0	0	Complex (3 and more chromosomal abnormalities)	adverse
11H104	NA	0	NA	inv(16)(p13.1q22)/t(16;16)(p13.1;q22)/CBFB-MYH11 (Irrespective of additional cytogenetic abnormalities)	favorable
11H107	0	0	0	t(8;21)(q22;q22)/RUNX1-RUNX1T1 (Irrespective of additional cytogenetic abnormalities)	favorable
11H126	1	0	1	Normal karyotype	intermediate
11H127	0	0	0	Complex (3 and more chromosomal abnormalities)	adverse

11H129	0	0	1	Intermediate abnormal karyotype (except isolated trisomy/tetrasomy 8)	intermediate
11H132	0	0	0	Complex (3 and more chromosomal abnormalities)	adverse
11H135	1	1	0	Normal karyotype	intermediate
11H138	0	1	0	Hyperdiploid numerical abnormalities only	Undetermined
11H140	0	0	0	Complex (3 and more chromosomal abnormalities)	adverse
11H142	1	1	0	Normal karyotype	intermediate
11H145	0	0	0	Complex (3 and more chromosomal abnormalities)	adverse
11H151	1	0	1	Normal karyotype	intermediate
11H157	1	1	1	Normal karyotype	intermediate
11H160	0	1	0	NUP98-NSD1(normal karyotype)	intermediate
11H170	0	0	0	Complex (3 and more chromosomal abnormalities)	adverse
11H175	0	0	0	Complex (3 and more chromosomal abnormalities)	adverse
11H177	0	0	0	Complex (3 and more chromosomal abnormalities)	adverse
11H179	0	0	0	inv(16)(p13.1q22)/t(16;16)(p13.1;q22)/CBFB-MYH11 (Irrespective of additional cytogenetic abnormalities)	favorable
11H182	0	1	0	Normal karyotype	intermediate
11H183	0	1	0	Intermediate abnormal karyotype (except isolated trisomy/tetrasomy 8)	intermediate
11H186	1	1	1	Normal karyotype	intermediate
11H187	0	0	0	Complex (3 and more chromosomal abnormalities)	adverse
11H192	0	0	0	Intermediate abnormal karyotype (except isolated trisomy/tetrasomy 8)	intermediate
11H194	0	0	0	Complex (3 and more chromosomal abnormalities)	adverse
11H195	1	0	0	Normal karyotype	intermediate
11H205	0	0	0	MECOM rearranged	adverse
11H217	1	0	0	Intermediate abnormal karyotype (except isolated trisomy/tetrasomy 8)	intermediate
11H220	1	0	0	Normal karyotype	intermediate
11H230	1	0	1	Intermediate abnormal karyotype (except isolated trisomy/tetrasomy 8)	intermediate
11H231	1	1	1	Normal karyotype	intermediate
11H232	0	0	0	Monosomy 5/ 5q-/Monosomy 7/ 7q- (less than 3 chromosomal abnormalities)	intermediate
11H234	1	1	0	Normal karyotype	intermediate
11H240	0	0	1	Intermediate abnormal karyotype (except isolated trisomy/tetrasomy 8)	intermediate
12H007	1	1	1	Normal karyotype	intermediate
12H010	1	1	1	Normal karyotype	intermediate
12H012	0	0	0	Complex (3 and more chromosomal abnormalities)	adverse
12H019	0	0	0	Complex (3 and more chromosomal abnormalities)	adverse
12H021	0	1	0	Normal karyotype	intermediate
12H030	0	0	0	Intermediate abnormal karyotype (except isolated trisomy/tetrasomy 8)	intermediate
12H033	1	1	0	Normal karyotype	intermediate
12H039	0	0	0	Intermediate abnormal karyotype (except isolated trisomy/tetrasomy 8)	intermediate
12H042	0	0	0	inv(16)(p13.1q22)/t(16;16)(p13.1;q22)/CBFB-MYH11 (Irrespective of additional cytogenetic abnormalities)	favorable
12H044	0	0	0	inv(16)(p13.1q22)/t(16;16)(p13.1;q22)/CBFB-MYH11 (Irrespective of additional cytogenetic abnormalities)	favorable
12H045	0	0	0	t(8;21)(q22;q22)/RUNX1-RUNX1T1 (Irrespective of additional cytogenetic abnormalities)	favorable
12H053	1	0	1	Normal karyotype	intermediate
12H055	0	0	0	Complex (3 and more chromosomal abnormalities)	adverse
12H056	1	1	1	Normal karyotype	intermediate
12H057	NA	0	NA	KMT2A rearranged	adverse
12H058	0	0	0	Complex (3 and more chromosomal abnormalities)	adverse
12H067	0	0	0	Complex (3 and more chromosomal abnormalities)	adverse
12H077	0	0	0	Complex (3 and more chromosomal abnormalities)	adverse
12H079	1	0	0	Intermediate abnormal karyotype (except isolated trisomy/tetrasomy 8)	intermediate
12H086	1	0	1	Normal karyotype	intermediate
12H091	1	0	0	Normal karyotype	intermediate
12H096	NA	0	NA	Complex (3 and more chromosomal abnormalities)	adverse
12H098	0	0	0	t(8;21)(q22;q22)/RUNX1-RUNX1T1 (Irrespective of additional cytogenetic abnormalities)	favorable
12H106	0	0	0	Complex (3 and more chromosomal abnormalities)	adverse
12H116	1	0	1	Normal karyotype	intermediate
12H117	0	0	1	Complex (3 and more chromosomal abnormalities)	adverse
12H119	0	0	1	Normal karyotype	intermediate
12H124	1	0	1	Normal karyotype	intermediate
12H132	1	1	1	Normal karyotype	intermediate
12H138	0	0	0	Complex (3 and more chromosomal abnormalities)	adverse
12H139	0	1	0	t(15;17)(q24;q21)/PML-RARA (Irrespective of additional cytogenetic abnormalities)	favorable
12H141	1	0	1	Normal karyotype	intermediate
12H148	0	0	0	MECOM rearranged	adverse
12H149	NA	0	NA	Complex (3 and more chromosomal abnormalities)	adverse
12H151	0	0	0	Intermediate abnormal karyotype (except isolated trisomy/tetrasomy 8)	intermediate
12H159	0	0	0	Complex (3 and more chromosomal abnormalities)	adverse
12H165	0	0	0	inv(16)(p13.1q22)/t(16;16)(p13.1;q22)/CBFB-MYH11 (Irrespective of additional cytogenetic abnormalities)	favorable
12H166	0	0	0	t(8;21)(q22;q22)/RUNX1-RUNX1T1 (Irrespective of additional cytogenetic abnormalities)	favorable
12H170	0	0	1	Complex (3 and more chromosomal abnormalities)	adverse
12H171	1	1	1	Normal karyotype	intermediate
12H172	0	0	0	Intermediate abnormal karyotype (except isolated trisomy/tetrasomy 8)	intermediate
12H173	1	0	0	Intermediate abnormal karyotype (except isolated trisomy/tetrasomy 8)	intermediate
12H175	0	0	0	Normal karyotype	intermediate
12H176	1	0	0	Intermediate abnormal karyotype (except isolated trisomy/tetrasomy 8)	intermediate
12H180	0	1	0	t(8;21)(q22;q22)/RUNX1-RUNX1T1 (Irrespective of additional cytogenetic abnormalities)	favorable
12H183	0	0	0	t(8;21)(q22;q22)/RUNX1-RUNX1T1 (Irrespective of additional cytogenetic abnormalities)	favorable
12H195	1	1	1	Normal karyotype	intermediate
13H006	0	0	0	Complex (3 and more chromosomal abnormalities)	adverse
13H009	0	0	0	Trisomy/tetrasomy 8 (isolated)	intermediate
13H012	0	0	0	Intermediate abnormal karyotype (except isolated trisomy/tetrasomy 8)	intermediate
13H018	0	0	0	Complex (3 and more chromosomal abnormalities)	adverse

13H019	1	1	0	Normal karyotype	intermediate
13H039	0	0	0	Intermediate abnormal karyotype (except isolated trisomy/tetrasomy 8)	intermediate
13H046	1	1	1	Normal karyotype	intermediate
13H048	0	0	0	Normal karyotype	intermediate
13H053	1	0	0	Normal karyotype	intermediate
13H056	0	0	0	Complex (3 and more chromosomal abnormalities)	adverse
13H058	0	0	0	Normal karyotype	intermediate
13H060	0	1	0	Normal karyotype	intermediate
13H064	0	0	0	Normal karyotype	intermediate
13H065	0	0	0	Intermediate abnormal karyotype (except isolated trisomy/tetrasomy 8)	intermediate
13H066	0	0	0	inv(16)(p13.1q22)/t(16;16)(p13.1;q22)/CBFB-MYH11 (Irrespective of additional cytogenetic abnormalities)	favorable
13H071	0	0	1	t(15;17)(q24;q21)/PML-RARA (Irrespective of additional cytogenetic abnormalities)	favorable
13H073	1	1	0	Normal karyotype	intermediate
13H078	0	1	0	t(15;17)(q24;q21)/PML-RARA (Irrespective of additional cytogenetic abnormalities)	favorable
13H080	0	0	1	Intermediate abnormal karyotype (except isolated trisomy/tetrasomy 8)	intermediate
13H083	0	0	0	MECOM rearranged	adverse
13H084	0	0	0	Normal karyotype	intermediate
13H100	NA	0	NA	Intermediate abnormal karyotype (except isolated trisomy/tetrasomy 8)	intermediate
13H101	NA	1	NA	t(6;9)(p23;q34) (Irrespective of additional cytogenetic abnormalities)	adverse
13H104	0	0	0	t(15;17)(q24;q21)/PML-RARA (Irrespective of additional cytogenetic abnormalities)	favorable
13H107	0	0	0	Complex (3 and more chromosomal abnormalities)	adverse
13H110	1	1	0	Normal karyotype	intermediate
13H114	1	1	0	Normal karyotype	intermediate
13H118	1	0	1	Normal karyotype	intermediate
13H120	0	1	0	inv(16)(p13.1q22)/t(16;16)(p13.1;q22)/CBFB-MYH11 (Irrespective of additional cytogenetic abnormalities)	favorable
13H126	0	1	0	t(15;17)(q24;q21)/PML-RARA (Irrespective of additional cytogenetic abnormalities)	favorable
13H132	0	0	0	Normal karyotype	intermediate
13H139	1	1	0	Intermediate abnormal karyotype (except isolated trisomy/tetrasomy 8)	intermediate
13H140	0	0	0	inv(16)(p13.1q22)/t(16;16)(p13.1;q22)/CBFB-MYH11 (Irrespective of additional cytogenetic abnormalities)	favorable
13H141	0	0	0	Complex (3 and more chromosomal abnormalities)	adverse
13H150	0	0	1	Complex (3 and more chromosomal abnormalities)	adverse
13H158	0	0	0	Intermediate abnormal karyotype (except isolated trisomy/tetrasomy 8)	intermediate
13H159	1	1	1	Intermediate abnormal karyotype (except isolated trisomy/tetrasomy 8)	intermediate
13H162	0	1	0	t(15;17)(q24;q21)/PML-RARA (Irrespective of additional cytogenetic abnormalities)	favorable
13H166	1	0	1	Normal karyotype	intermediate
13H169	0	0	0	t(8;21)(q22;q22)/RUNX1-RUNX1T1 (Irrespective of additional cytogenetic abnormalities)	favorable
13H170	0	1	0	Normal karyotype	intermediate
13H173	1	0	1	Normal karyotype	intermediate
13H179	0	0	0	Intermediate abnormal karyotype (except isolated trisomy/tetrasomy 8)	intermediate
13H182	1	0	1	Normal karyotype	intermediate
13H185	0	0	1	Intermediate abnormal karyotype (except isolated trisomy/tetrasomy 8)	intermediate
13H186	1	1	1	Intermediate abnormal karyotype (except isolated trisomy/tetrasomy 8)	intermediate
14H001	1	1	0	Normal karyotype	intermediate
14H003	1	1	0	Normal karyotype	intermediate
14H007	1	1	1	Normal karyotype	intermediate
14H012	0	0	0	Intermediate abnormal karyotype (except isolated trisomy/tetrasomy 8)	intermediate
14H015	1	1	1	Normal karyotype	intermediate
14H017	0	0	0	Intermediate abnormal karyotype (except isolated trisomy/tetrasomy 8)	intermediate
14H018	NA	1	NA	t(15;17)(q24;q21)/PML-RARA (Irrespective of additional cytogenetic abnormalities)	favorable
14H019	0	0	1	Normal karyotype	intermediate
14H020	1	0	0	Normal karyotype	intermediate
14H023	0	0	1	MECOM rearranged	adverse
14H024	0	0	0	Monosomy 5/ 5q-/Monosomy 7/ 7q- (less than 3 chromosomal abnormalities)	adverse
14H026	1	1	1	Normal karyotype	intermediate
14H027	0	0	0	Intermediate abnormal karyotype (except isolated trisomy/tetrasomy 8)	intermediate
14H028	1	1	0	Normal karyotype	intermediate
14H031	0	0	0	KMT2A rearranged	adverse
14H038	0	0	0	MECOM rearranged	adverse
14H049	0	0	1	Normal karyotype	intermediate
14H051	1	0	1	Normal karyotype	intermediate
14H053	0	1	0	KMT2A rearranged	intermediate
14H059	0	0	1	Monosomy 5/ 5q-/Monosomy 7/ 7q- (less than 3 chromosomal abnormalities)	intermediate
14H071	NA	0	NA	t(15;17)(q24;q21)/PML-RARA (Irrespective of additional cytogenetic abnormalities)	favorable
14H089	1	1	0	Normal karyotype	intermediate
14H093	0	1	1	Normal karyotype	intermediate
14H094	0	0	0	Complex (3 and more chromosomal abnormalities)	adverse
14H095	1	1	1	Normal karyotype	intermediate
14H102	0	0	0	KMT2A rearranged	adverse
14H103	0	0	0	inv(16)(p13.1q22)/t(16;16)(p13.1;q22)/CBFB-MYH11 (Irrespective of additional cytogenetic abnormalities)	favorable
14H109	1	1	1	Normal karyotype	intermediate
14H112	1	1	0	Normal karyotype	intermediate
14H119	0	0	0	Complex (3 and more chromosomal abnormalities)	adverse
14H124	0	0	0	Intermediate abnormal karyotype (except isolated trisomy/tetrasomy 8)	intermediate
14H126	0	0	0	Intermediate abnormal karyotype (except isolated trisomy/tetrasomy 8)	intermediate
14H133	0	1	0	t(15;17)(q24;q21)/PML-RARA (Irrespective of additional cytogenetic abnormalities)	favorable
14H143	0	1	0	Normal karyotype	intermediate
14H145	1	1	0	Normal karyotype	intermediate
14H151	1	0	1	Normal karyotype	intermediate
14H155	1	0	0	Normal karyotype	intermediate
14H156	1	1	1	Normal karyotype	intermediate

14H164	1	1	0	Intermediate abnormal karyotype (except isolated trisomy/tetrasomy 8)	intermediate
14H171	1	1	1	Normal karyotype	intermediate
14H172	0	1	0	Normal karyotype	intermediate
15H002	0	0	0	Trisomy/tetrasomy 8 (isolated)	intermediate
15H003	0	0	0	KMT2A rearranged	intermediate
15H013	0	1	1	Normal karyotype	intermediate
15H023	0	1	0	Normal karyotype	intermediate
15H027	0	0	1	Complex (3 and more chromosomal abnormalities)	adverse
15H033	0	0	0	Normal karyotype	intermediate
15H036	1	0	1	Normal karyotype	intermediate
15H037	0	0	0	Complex (3 and more chromosomal abnormalities)	adverse
15H038	0	0	0	Monosomy 5/ 5q-/Monosomy 7/ 7q- (less than 3 chromosomal abnormalities)	intermediate
15H044	1	1	1	Normal karyotype	intermediate
15H045	0	0	0	Monosomy 5/ 5q-/Monosomy 7/ 7q- (less than 3 chromosomal abnormalities)	adverse
15H049	0	1	0	NUP98 translocations (+NUP98 FISH positive) (Irrespective of additional cytogenetic abnormalities)	intermediate
15H058	1	0	0	Complex (3 and more chromosomal abnormalities)	adverse
15H059	0	1	0	Normal karyotype	intermediate
15H061	1	1	1	Normal karyotype	intermediate
15H062	0	1	0	Normal karyotype	intermediate
15H063	0	1	0	Trisomy/tetrasomy 8 (isolated)	intermediate
15H065	0	0	0	Complex (3 and more chromosomal abnormalities)	adverse
15H066	0	0	0	Intermediate abnormal karyotype (except isolated trisomy/tetrasomy 8)	intermediate
15H068	1	0	0	Normal karyotype	intermediate
15H071	0	1	0	Intermediate abnormal karyotype (except isolated trisomy/tetrasomy 8)	intermediate
15H080	0	0	0	Complex (3 and more chromosomal abnormalities)	adverse
15H082	1	0	0	Normal karyotype	intermediate
15H083	1	1	0	Normal karyotype	intermediate
15H085	0	1	0	Normal karyotype	intermediate
15H090	1	0	0	Normal karyotype	intermediate
15H103	1	1	0	Intermediate abnormal karyotype (except isolated trisomy/tetrasomy 8)	intermediate
15H105	1	0	1	Trisomy/tetrasomy 8 (isolated)	intermediate
15H106	1	1	0	Normal karyotype	intermediate
15H119	1	0	1	Intermediate abnormal karyotype (except isolated trisomy/tetrasomy 8)	intermediate
15H122	1	1	1	Intermediate abnormal karyotype (except isolated trisomy/tetrasomy 8)	intermediate
15H135	1	0	0	Normal karyotype	intermediate
15H138	1	0	0	Intermediate abnormal karyotype (except isolated trisomy/tetrasomy 8)	intermediate
15H139	1	1	1	Intermediate abnormal karyotype (except isolated trisomy/tetrasomy 8)	intermediate
15H144	0	0	0	Complex (3 and more chromosomal abnormalities)	adverse
16H007	0	0	0	Complex (3 and more chromosomal abnormalities)	adverse
16H008	1	0	0	Normal karyotype	intermediate
16H011	1	1	0	Normal karyotype	intermediate
16H013	1	0	1	Normal karyotype	intermediate
16H015	1	1	1	Normal karyotype	intermediate
16H018	0	0	0	Intermediate abnormal karyotype (except isolated trisomy/tetrasomy 8)	intermediate
16H027	0	0	0	Complex (3 and more chromosomal abnormalities)	adverse
16H042	0	0	1	Complex (3 and more chromosomal abnormalities)	adverse
16H044	0	0	1	Intermediate abnormal karyotype (except isolated trisomy/tetrasomy 8)	intermediate
16H045	0	1	0	Complex (3 and more chromosomal abnormalities)	adverse
16H063	0	1	0	KMT2A rearranged	adverse
16H064	0	1	0	Normal karyotype	intermediate
16H073	NA	0	NA	KMT2A rearranged	adverse
16H088	1	0	1	Normal karyotype	intermediate
16H089	0	0	0	Normal karyotype	intermediate
16H093	1	0	1	Normal karyotype	intermediate
16H097	0	0	0	Intermediate abnormal karyotype (except isolated trisomy/tetrasomy 8)	intermediate
16H098	0	0	0	Normal karyotype	intermediate
16H099	0	0	0	Normal karyotype	intermediate
16H100	1	1	0	Normal karyotype	intermediate
16H105	0	0	0	Normal karyotype	intermediate
16H106	1	1	1	Normal karyotype	intermediate
16H107	0	1	0	Normal karyotype	intermediate
16H108	1	0	0	Normal karyotype	intermediate
16H112	1	1	0	Normal karyotype	intermediate
16H115	0	0	0	Normal karyotype	intermediate
16H119	1	0	0	Intermediate abnormal karyotype (except isolated trisomy/tetrasomy 8)	intermediate
16H129	0	0	1	Normal karyotype	intermediate
16H132	1	0	1	Normal karyotype	intermediate
16H139	1	0	0	Normal karyotype	intermediate
16H142	1	1	1	Normal karyotype	intermediate
16H144	NA	0	NA	Complex (3 and more chromosomal abnormalities)	adverse
16H148	1	1	1	Normal karyotype	intermediate
17H008	0	0	0	KMT2A rearranged	adverse
17H013	1	0	1	Normal karyotype	intermediate
17H023	1	1	1	Normal karyotype	intermediate
17H027	1	0	0	Normal karyotype	intermediate
17H033	1	0	1	Normal karyotype	intermediate
17H036	1	1	1	Intermediate abnormal karyotype (except isolated trisomy/tetrasomy 8)	intermediate
17H037	0	0	0	KMT2A rearranged	adverse
17H043	0	0	0	NUP98 translocations (+NUP98 FISH positive) (Irrespective of additional cytogenetic abnormalities)	adverse
17H045	1	0	0	Intermediate abnormal karyotype (except isolated trisomy/tetrasomy 8)	intermediate

17H059	0	1	1	Trisomy/tetrasomy 8 (isolated)	intermediate
17H064	1	1	1	Normal karyotype	intermediate
17H065	0	0	1	Monosomy 5/ 5q-/Monosomy 7/ 7q- (less than 3 chromosomal abnormalities)	adverse
17H073	1	1	0	Normal karyotype	intermediate
17H075	0	0	0	Complex (3 and more chromosomal abnormalities)	adverse
17H082	1	1	0	Normal karyotype	intermediate
17H093	1	1	1	Complex (3 and more chromosomal abnormalities)	adverse
17H105	0	0	0	MECOM rearranged	adverse
17H125	0	0	0	Normal karyotype	intermediate
17H154	0	0	0	MECOM rearranged	adverse
17H155	1	0	0	Intermediate abnormal karyotype (except isolated trisomy/tetrasomy 8)	intermediate
17H167	0	0	0	Trisomy/tetrasomy 8 (isolated)	intermediate
17H176	1	0	0	Normal karyotype	intermediate
17H177	1	0	1	Normal karyotype	intermediate
17H209	0	0	0	Complex (3 and more chromosomal abnormalities)	adverse
17H210	NA	0	NA	MECOM rearranged	adverse
18H006	1	0	0	Normal karyotype	intermediate
18H016	0	0	0	Normal karyotype	intermediate
18H027	0	0	0	Complex (3 and more chromosomal abnormalities)	adverse
18H030	0	0	1	Intermediate abnormal karyotype (except isolated trisomy/tetrasomy 8)	intermediate
18H045	1	1	0	Normal karyotype	intermediate
18H047	0	0	1	Complex (3 and more chromosomal abnormalities)	adverse
18H049	NA	0	NA	KMT2A rearranged	adverse
18H053	1	0	1	Normal karyotype	intermediate
18H058	1	1	1	Normal karyotype	intermediate
18H062	1	1	1	Normal karyotype	intermediate
18H063	1	0	1	Normal karyotype	intermediate
18H072	NA	0	NA	KMT2A rearranged	adverse
18H089	1	1	1	Normal karyotype	intermediate
18H093	1	1	1	Normal karyotype	intermediate
18H094	0	0	0	MECOM rearranged	adverse
18H104	1	1	0	Normal karyotype	intermediate
18H118	0	0	1	Monosomy 5/ 5q-/Monosomy 7/ 7q- (less than 3 chromosomal abnormalities)	intermediate
18H119	1	1	0	Normal karyotype	intermediate
18H131	0	0	0	Intermediate abnormal karyotype (except isolated trisomy/tetrasomy 8)	intermediate
18H137	1	1	1	Normal karyotype	intermediate
18H138	1	1	1	Normal karyotype	intermediate
18H141	1	0	1	Normal karyotype	intermediate
18H146	0	0	1	Monosomy 5/ 5q-/Monosomy 7/ 7q- (less than 3 chromosomal abnormalities)	adverse
18H151	0	0	1	Normal karyotype	intermediate
18H152	0	0	1	Monosomy 5/ 5q-/Monosomy 7/ 7q- (less than 3 chromosomal abnormalities)	adverse
18H156	0	1	0	Intermediate abnormal karyotype (except isolated trisomy/tetrasomy 8)	intermediate
18H166	0	0	0	Intermediate abnormal karyotype (except isolated trisomy/tetrasomy 8)	intermediate
18H175	1	0	0	Normal karyotype	intermediate
18H182	0	1	0	Normal karyotype	intermediate
18H194	0	0	0	Normal karyotype	intermediate
18H195	0	0	0	Complex (3 and more chromosomal abnormalities)	adverse
18H206	1	0	0	Normal karyotype	intermediate
18H207	1	1	1	Normal karyotype	intermediate
18H208	0	0	1	Normal karyotype	intermediate
19H045	0	0	0	Germ cell tumor -associated hematologic malignancies_iso12p	adverse

Supplement Table 4: Leucegene AML cohort NPM1, DNMT3A, FLT3-ITD mutation and cytogenetic subgroup and risk.

Key	Value	P_val	Q_val	Odds	Selected	Total
tissue	Blood	3.60E-13	1.40E-11	4.3	102/319	691
WBC lt 20	>=20	8.70E-09	1.60E-07	3.3	108/390	687
cytogenetic_group	t(8;21)(q22;q22)/RUNX1-RUNX1T1 (Irrespective of additional cytogenetic abnormalities)	1.10E-07	7.70E-07	13.2	15/20	691
subgroup	t(8;21)(q22;q22)/RUNX1-RUNX1T1 (Irrespective of additional cytogenetic abnormalities)	1.10E-07	7.70E-07	13.2	15/20	691
cytogenetic_risk	favorable	1.30E-07	7.70E-07	3.8	36/82	691
mutation FLT3_VALID	1	7.10E-07	3.60E-06	2.7	64/197	659
fab	AML-M3V	2.90E-04	1.20E-03	14.6	09-juil	691
WBC lt 100	>=100	5.00E-04	1.70E-03	2.2	37/114	687
cytogenetic_group	t(15;17)(q24;q21)/PML-RARA (Irrespective of additional cytogenetic abnormalities)	7.20E-04	2.20E-03	3.8	14/30	691
subgroup	t(15;17)(q24;q21)/PML-RARA (Irrespective of additional cytogenetic abnormalities)	7.20E-04	2.20E-03	3.8	14/30	691
transcriptome_protocol	non-stranded	2.00E-03	3.90E-03	1.9	49/174	691
mutation TP53_VALID	0	2.00E-03	4.10E-03	3.2	128/584	659
mutation NRAS_VALID	0	3.90E-03	6.20E-03	2.5	124/562	659
mutation KIT_VALID	1	4.10E-03	6.20E-03	3	13/31	659
del5q_WGS	0	4.70E-03	6.20E-03	4.3	94/362	402
mutation SRSF2_VALID	0	5.70E-03	6.20E-03	3.1	129/597	659
mutation NF1_VALID	0	5.80E-03	6.20E-03	5.1	132/619	659
mutation BCOR_VALID	0	1.30E-02	1.30E-02	7.2	133/631	659
mutation STAG2_VALID	0	1.50E-02	1.50E-02	4.4	132/624	659
mutation ASXL2_VALID	1	1.80E-02	1.80E-02	8	06-avr	659
mutation ZRSR2_VALID	1	1.80E-02	1.80E-02	8	06-avr	659
status at sampling	Relapse/Refractory	2.20E-02	2.20E-02	2	18/57	691
mutation WT1_VALID	1	3.40E-02	3.40E-02	1.8	20/67	659
mutation CEBPA_VALID	0	3.50E-02	3.50E-02	2.7	130/615	659
mutation NPM1_VALID	1	3.80E-02	3.80E-02	1.4	59/244	659

Supplement Table 5: Clinical characteristic enrichment of 20% of sample with highest expression of IL1RAP.
